# CAPG and GIPC1: Breast Cancer Biomarkers for Bone Metastasis Development and Treatment

**DOI:** 10.1093/jnci/djv360

**Published:** 2016-01-12

**Authors:** Jules A. Westbrook, David A. Cairns, Jianhe Peng, Valerie Speirs, Andrew M. Hanby, Ingunn Holen, Steven L. Wood, Penelope D. Ottewell, Helen Marshall, Rosamonde E. Banks, Peter J. Selby, Robert E. Coleman, Janet E. Brown

**Affiliations:** **Affiliations of authors:**Academic Unit of Clinical Oncology, University of Sheffield, Sheffield, UK (JAW*, IH, PDO, REC, JEB*); Cancer Research UK Leeds Centre (JAW, DAC, JP, SLW, REB, PJS, JEB), Clinical Trials Research Unit, Leeds Institute of Clinical Trials Research (DAC*, HM), and Clinical and Biomedical Proteomics Group (JAW, DAC, JP, SLW, REB, PJS, JEB) and Pathology and Tumor Biology (VS, AMH), Leeds Institute of Cancer and Pathology, University of Leeds, UK; Department of Oncology and Metabolism, University of Sheffield, Sheffield, UK (SLW*).

## Abstract

**Background:**

Bone is the predominant site of metastasis from breast cancer, and recent trials have demonstrated that adjuvant bisphosphonate therapy can reduce bone metastasis development and improve survival. There is an unmet need for prognostic and predictive biomarkers so that therapy can be appropriately targeted.

**Methods:**

Potential biomarkers for bone metastasis were identified using proteomic comparison of bone-metastatic, lung-metastatic, and nonmetastatic variants of human breast cancer MDA-MB-231 cells. Clinical validation was performed using immunohistochemical staining of tumor tissue microarrays from patients in a large randomized trial of adjuvant zoledronic acid (zoledronate) (AZURE-ISRCTN79831382). We used Cox proportional hazards regression, the Kaplan-Meier estimate of the survival function, and the log-rank test to investigate associations between protein expression, clinical variables, and time to distant recurrence events. All statistical tests were two-sided.

**Results:**

Two novel biomarker candidates, macrophage-capping protein (CAPG) and PDZ domain–containing protein GIPC1 (GIPC1), were identified for clinical validation. Cox regression analysis of AZURE training and validation sets showed that control patients (no zoledronate) were more likely to develop first distant recurrence in bone (hazard ratio [HR] = 4.5, 95% confidence interval [CI] = 2.1 to 9.8, *P* < .001) and die (HR for overall survival = 1.8, 95% CI = 1.01 to 3.24, *P* = .045) if both proteins were highly expressed in the primary tumor. In patients with high expression of both proteins, zoledronate had a substantial effect, leading to 10-fold hazard ratio reduction (compared with control) for first distant recurrence in bone (*P* = .008).

**Conclusions:**

The composite biomarker, CAPG and GIPC1 in primary breast tumors, predicted disease outcomes and benefit from zoledronate and may facilitate patient selection for adjuvant bisphosphonate treatment.

The skeleton is the predominant site for metastasis in breast cancer, providing a fertile microenvironment for survival and growth of disseminated tumor cells. Bone-targeted agents such as bisphosphonates and denosumab, which disrupt the destructive interactions between cancer and bone cells, are widely used to prevent skeletal complications of bone metastasis but have also been investigated as adjuvant agents to prevent or delay bone metastasis. Several large trials of adjuvant bisphosphonate therapy in early breast cancer have now reported (1–3), including the open-label, multicenter, phase III AZURE trial (BIG01/04 - ISRCTN79831382), which recruited 3360 patients with stage II/III breast cancer randomized (1:1) to standard adjuvant therapy alone (control) or standard therapy with zoledronic acid (zoledronate) (19 doses of 4mg in 5 years) (1). After a median of 84 months follow-up, for the whole trial population, although there was no statistical difference in disease-free survival, zoledronate reduced bone metastases (adjusted hazard ratio [HR] = 0.78, 95% confidence interval [CI] = 0.63 to 0.96, *P* = .020). Moreover, in a preplanned analysis, zoledronate improved disease outcomes for women (n = 1041) who were more than five years postmenopausal at diagnosis (adjusted HR for invasive disease-free survival = 0.77, 95% CI = 0.63 to 0.96) (4). Furthermore, a meta-analysis of individual patient data in 17 791 women from 22 randomized trials confirmed that in postmenopausal women adjuvant bisphosphonates reduced bone recurrences and breast cancer death by 34% (*P* < .001) and 17% (*P* = .004), respectively (5). These studies are likely to be practice changing but also highlight the unmet need for biomarkers to identify patients at risk of bone metastasis to guide selection for adjuvant bisphosphonate treatment.

In the past decade, multiple gene expression datasets from analysis of breast cancer metastasis have identified key pathways underlying determinants of metastasis and provided information on which genes drive metastasis to specific organs, including the skeleton (6–11). Proteomic approaches also have high potential for the development of biomarkers for prediction of metastasis development (12).

In this study, we have identified novel bone metastasis-associated biomarkers from proteomics studies in cell lines, verified the increased expression of these proteins in bone homing cells, and carried out clinical validation in large training and independent validation sets on tissue microarrays (TMAs) from patients in the AZURE study, leading to a clinically validated composite biomarker with both prognostic and predictive utility.

## Methods

### Proteomic Analysis and Identification of Candidate Biomarkers

**Figure 1.**
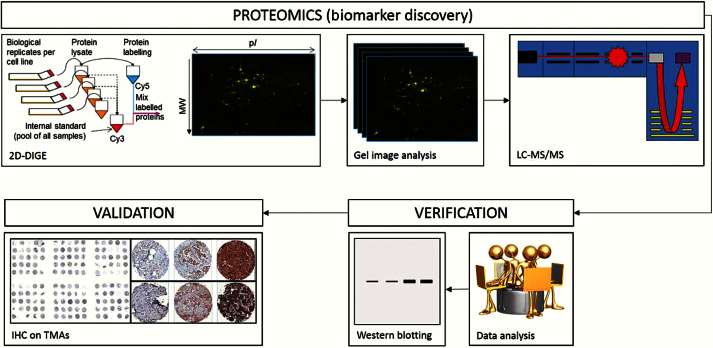
Simplified flow diagram showing key proteomic steps used for discovery of novel biomarkers for risk of bone metastasis development. Proteins extracted from multiple independent cultures of cell lines were labeled using Cy5 fluorescent dye while an internal standard was labeled with Cy3. Separation of proteins (by isoelectric point and molecular weight) and image capture (fluoresence densitometry) creates protein array images that can be compared so as to detect differential protein expression between cell line types and replicates (intensity of fluoresence). Proteins of higher expression in bone-homed cell lines were excised from silver-stained two-dimensional difference gel electrophoresis gels, reduced to peptides, and analyzed using tandem mass spectrometry. Identified proteins were assessed for known/reported relevance to breast cancer and/or bone metastasis prior to selection for validation of expression on breast cancer tissue microarrays. 2D-DIGE = two-dimensional difference gel electrophoresis; IHC = immunohistochemistry; LC/MS/MS = liquid chromatography/mass spectrometry/mass spectrometry; TMA = tissue microarray.

Metastatic variants of the human breast cancer cell line MDA-MB-231 home to bone (BM1, BM2) or lung (LM), when administered intravenously to nude mice, whereas the ‘parental’ MDA-MB-231 cells (PCC) do not (8,13). We explored differences in the proteomes of BM1, BM2, LM, and PCC cells to identify differentially regulated proteins specifically associated with development of bone metastases in human breast cancer. Figure 1 indicates the key steps in our approach for the proteomic discovery of novel biomarkers.

Differential protein expression between multiple independent cultures of these cell lines was quantified using two-dimensional difference gel electrophoresis (2D-DIGE) (14). Following analysis of the 2D-DIGE gels, gel spots of interest were excised manually from silver-stained DIGE gels, and tryptic peptides were generated for mass spectrometric analysis using an in-gel digestion method. Proteins were identified using nano–liquid chromatography/mass spectrometry/mass spectrometry (LC/MS/MS) analysis on a QSTAR XL quadrupole time-of-flight hybrid mass spectrometer (AB Sciex, Warrington, UK) coupled online with an Agilent 1100 Series nano-LC System (Agilent Technologies, Berkshire, UK) through electrospray ionization. The MS/MS raw data files from the LC/MS/MS analysis were processed by Analyst v2.0 and a script plug-in Mascot.dll 1.6b24 (AB Sciex, Warrington, UK) then sent to the local Mascot database search engine (v2.3, Matrix Science, Boston, MA).

Proteins with statistically significantly higher expression relative to PCC cells and greatest fold-change in the bone metastatic cell lines (fold changes ≥ 2, *P* = .029 by Wilcoxon-Mann-Whitney test), but not with higher expression in the LM cells, were assessed for relevance in cancer and/or bone metastasis using published literature and verified by western blotting. Further details are available in the Supplementary Materials (available online).

### Clinical Validation of Potential Biomarkers of Bone Metastasis Risk

#### Patients and Samples

All analyses on patient samples were performed with Ethics approval and informed patient consent. Initially, protein expression of target molecules was characterized using a local TMA constructed from 364 breast cancer samples graded 1, 2, and 3 (Data Supplement, available online). The main patient-based analyses were then performed on TMAs constructed from primary tumors from patients recruited into the AZURE trial (1). This provides an excellent resource for validation of protein biomarkers emerging from our proteomics studies because of the relatively high prevalence of bone metastatic outcomes and long follow-up (median = 84 months, interquartile range = 66–93). Triplicate cores of breast tumor tissue were arrayed across replicate TMAs for immunohistochemistry.

#### Immunohistochemistry

Protein expression was assessed on TMAs using immunohistochemistry (15,16). Briefly, 5 µm serial sections of TMA were dewaxed in xylene and rehydrated through graded alcohols. Endogenous peroxidases were blocked (3% H_2_0_2_, 10 minutes), and antigens retrieved by microwaving slides. After cooling and washing, slides were blocked with goat serum (1:10; Zymed antibody diluent; 20 minutes), after which primary antibodies were applied (overnight, 4°C). Details of primary and secondary antibodies are presented in Supplementary Table 9 (available online). Following washing and incubation with HRP-conjugated secondary antibodies, proteins were visualized using diaminobenzidine before counterstaining with haematoxylin, dehydration, and mounting.

A three-tier ordinal categorical system was used to rank the tumors based on intensity of cytoplasmic staining (15,16), where 1 = weak staining; 2 = moderate, easily perceived staining; 3 = strong/intense staining; ie, the scoring was based on staining intensity only and not on percentage of positivity (Supplementary Materials, available online). In analyses, for simplification, the term ‘high’ refers to a staining score of 3, and ‘low’ to scores of 1 or 2. Cytoplasmic staining scores were assessed independently by two trained operators, blinded to outcome data, under the supervision of an experienced histopathologist (AMH), who also adjudicated discrepant scores and the level of agreement of the two scores was measured using Cohen’s kappa coefficient.

#### Statistical Analyses

Statistical analyses followed REMARK guidelines (17) and tested the associations between protein expression and all relevant clinical and pathological variables available (eg, estrogen receptor [ER]/progesterone receptor [PR]/human epidermal growth factor receptor 2 [HER2] status) using Fisher’s Exact test for categorical variables and the Kruskal-Wallis test (for continuous variables) (18), before assessing associations with time-to-event data (time to first distant recurrence, time to first skeletal recurrence, time to first nonskeletal recurrence) using Cox proportional hazards regression, the Kaplan-Meier estimate of the survival function, and the log-rank test. Time to first distant recurrence was defined as the time from the date of random assignment to the date of the distant recurrence. In analyses, other types of events were censored; eg, if a local recurrence occurred prior to any distant recurrence, the patient would be censored at the date of the local recurrence. Time to first skeletal recurrence and first nonskeletal recurrence were defined similarly. Time to first skeletal recurrence irrespective of all other previous recurrences was also investigated.

Time-to-event analysis was first performed within treatment arms to identify any prognostic effects related to the biomarkers. Subsequently, similar analyses were performed for the treatment effect within subgroups defined by biomarker status to assess predictive effects of the biomarker. The predictive heterogeneity of effect between treatment arms for time to distant events was assessed in multivariable analysis by including an interaction term in the Cox proportional hazard regressions for treatment arm and biomarker (while adjusting for systemic therapy plan, ER status, and lymph node involvement). All statistical tests were two-sided.

## Results

### Proteomic Studies, Selection of Proteins for Further Study, and Immunohistochemistry

**Figure 2.**
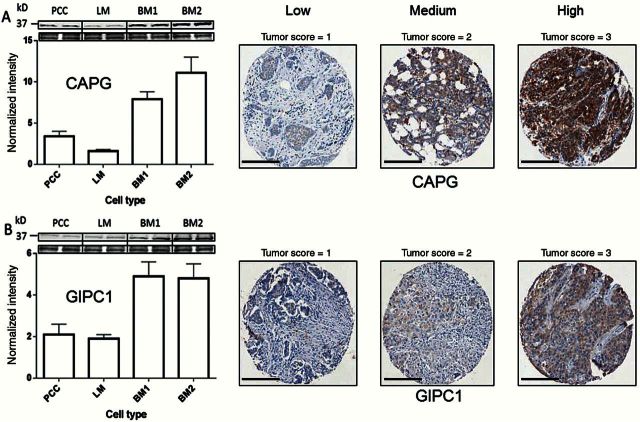
Macrophage-capping protein (CAPG) and PDZ domain–containing protein GIPC1 (GIPC1) protein expression in cells and patient breast cancer tissue. **Left hand panel** shows western blot verification of high CAPG and GIPC1 protein expression in BM1 and BM2 cell lysates compared with parental cells (expression changes >2-fold with *P* < .03, by Student’s *t* test); **upper part** shows blot expression with loading control. Data are means ± SD. **Right hand panel** illustrates examples of differential protein expression in AZURE tissue microarray breast tumor cores for CAPG and GIPC1. In each case, the antibody localization has been used in conjunction with diaminobenzidine (DAB; **brown**), and the scoring is based on the intensity of staining in the cytoplasmic compartment in the tumor cells only. **Scale bar** = 200 µm. CAPG = macrophage-capping protein; GIPC1 = PDZ domain–containing protein GIPC1; PCC = MDA-MB-231 cells.

Table 1 summarizes key proteomic results (further details in the Supplementary Materials, available online). Data were collected for 1292 2D-DIGE–resolved gel spots for comparative analyses. Principal components analysis demonstrated clustering of cell types with parental control cells separated from metastatic cell types. Bone metastatic variants clustered separately from parental cells and from lung metastatic variants, indicating that differences from parental cells are organ specific and not simply a general metastatic effect. Nearly 1000 gel spots demonstrated evidence of differential protein expression between cell types (*P* < .05, Kruskal-Wallis test [18]). In order to isolate gel spots with the most robust and statistically significant differential expression, stringent selection and filtering criteria were used (see the Supplementary Materials, available online). A list of 32 DIGE spots was determined, returning 75 unique protein identifications where fold changes were 2 or greater (*P* = .029, Wilcoxon-Mann-Whitney test). We focused on the eight of these proteins that were statistically significantly upregulated in the BM cell lines only. These were then assessed on the basis of highest confidence in identification by mass spectrometry and/or likely relevance to breast cancer and/or bone metastasis using published literature (see Table 1), and the expression of proteins selected on this basis was tested directly in BM1 and BM2 cells using western blotting (Figure 2). These were: macrophage-capping protein (CAPG); PDZ domain-containing protein GIPC1 (GIPC1); and transcriptional activator protein Pur-alpha (PURA). Western blotting showed that CAPG, GIPC1, and PURA antibodies (see Supplementary Table 9, available online) detected single bands (at the appropriate molecular weight for their respective antigens), demonstrating their specificity. Figure 2 shows that CAPG and GIPC1 (but not PURA – data not shown) were verified as having higher expression in BM cell lysates, and on this basis CAPG and GIPC1 were selected for clinical validation.

**Table 1.** Summary of key proteomic results*

DIGE spot#	Fold change	K-W *P*	Entry	Entry name	Protein name	Gene	Relevance to breast cancer and/or bone metastasis
751	2.2	.0039	P17987	TCPA_HUMAN	T-complex protein 1 subunit alpha	TCP1	Little or no relevant literature
904	2.0	.0028	P31943	HNRH1_HUMAN	Heterogeneous nuclear ribonucleoprotein H (hnRNP H)	HNRNPH1	Some literature, but not strong relevance (31)
904	2.0	.0028	Q96KP4	CNDP2_HUMAN	Cytosolic-nonspecific dipeptidase (EC 3.4.13.18)	CNDP2	Little or no relevant literature
1106	2.5	.0034	P06132	DCUP_HUMAN	Uroporphyrinogen decarboxylase (EC 4.1.1.37)	UROD	Some literature, but not strong relevance (32)
1106	2.5	.0034	P40121	CAPG_HUMAN	Macrophage-capping protein	CAPG	Significant relevant literature (33,34)
1106	2.5	.0034	P53365	ARFP2_HUMAN	Arfaptin-2	ARFIP2	Little or no relevant literature
1106	2.5	.0034	Q00577	PURA_HUMAN	Transcriptional activator protein Pur-alpha	PURA	Little or no relevant literature
1158	2.0	.0042	O14908	GIPC1_HUMAN	PDZ domain–containing protein GIPC1	GIPC1	Significant relevant literature (27,35–37)

* “Fold change” refers to the average of the relevant pair-wise comparisons possible within the dataset. Of the original 75 proteins identified with statistically significant fold changes, eight were upregulated in BM cells only (and no other cells) as shown in the table. These eight proteins were selected for further consideration by looking for literature evidence of relevance to breast cancer and/or bone metastasis as indicated in the table. K-W *P* value refers to the Kruskal-Wallis nonparametric analysis of variance test. Entry details from Universal Protein Resource (UniProt) tags are also shown. (NB multiple protein identifications from one two-dimensional gel electrophoresis spot are common.) DIGE = difference gel electrophoresis.

The CAPG and GIPC1 antibodies were then subsequently used for immunohistochemistry. The CAPG antibody is formally certified for immunohistochemistry (IHC) application (see Supplementary Table 9, available online). While the Abcam GIPC1 antibody is not formally certified, this is not unusual for some antibodies and in our hands it performed well under formalin-fixed, paraffin-embedded–IHC conditions. A single specific band at the appropriate molecular weight for GIPC1 was detected in WB analysis of cell lysates (36kDa) (Figure 2), providing confidence that the antibody is robust. Each antibody showed a wide range of cytoplasmic staining intensity in the graded breast cancer TMAs (see Supplementary Materials, available online), demonstrating appropriate antibody sensitivity. TMAs for the training and validation sets were stained a few months apart using antibodies from the same supplier, though for GIPC1 from two batches (lots). Nevertheless, identical staining profiles were observed for both TMA sets, which were validated by a specialist breast histopathologist (AMH), providing confidence on reproducibility.

### Patients

We initially explored associations between clinical outcomes and TMA immunohistochemistry scores for CAPG and GIPC1 (primary antibodies from Sigma HPA019080, rabbit IgG, and abcam ab89684, mouse IgG, respectively) in a training set of 427 randomly assigned AZURE trial patients (211 control, 216 zoledronate). A second independent validation set from 297 randomly assigned AZURE trial patients (147 control, 150 zoledronate) was available for confirmation of findings from the training set. There was a high level of agreement between the two independent scorers as judged by Cohen’s kappa score (overall, κ = 0.85 and κ = 0.80 for CAPG and GIPC1, respectively). Table 2 displays the patient characteristics of both training and validation sets and the combined sets and shows that these are similar to those of the overall AZURE population. In association analyses, neither CAPG nor GIPC1 expression showed any statistical association with baseline variables (eg, age, lymph node involvement, ER status, menopausal status, systemic therapy, chemotherapy, and statin use) (see Supplementary Tables 1, 2, and 3, available online).

**Table 2.** Characteristics of the patients whose tissue was assessed on TMAs in this study (as at baseline on the AZURE study) and first disease-free survival events

Characteristic	Training set		Validation set		Combined sets		Full AZURE trial population	
	Zoledronate (n = 216)	Control (n = 211)	Zoledronate (n = 147)	Control (n = 150)	Zoledronate (n = 363)	Control (n = 361)	Zoledronate (N = 1681)	Control (n = 1678)
Age, median (range), y	51 (26–75)	51 (33–79)	50 (26–77)	52 (33–79)	50 (26–77)	52 (33–79)	51 (20–87)	51 (21–89)
Axillary lymph nodes, No. (%)								
0	1 (0.5)	4 (1.9)	1 (0.7)	1 (0.7)	2 (0.6)	5 (1.4)	30 (1.8)	32 (1.9)
1–3	140 (64.8)	147 (69.7)	107 (72.8)	96 (64.0)	247 (68.0)	243 (67.3)	1042 (62.0)	1033 (61.6)
≥4	75 (34.7)	60 (28.4)	39 (26.5)	53 (35.3)	114 (31.4)	113 (31.3)	604 (35.9)	607 (36.2)
Tumor stage, No. (%)								
T1	73 (33.8)	80 (37.9)	55 (37.4)	58 (38.7)	128 (35.3)	138 (38.2)	542 (32.2)	523 (31.2)
T2	117 (54.2)	98 (46.4)	76 (51.7)	77 (51.3)	193 (53.2)	175 (48.5)	850 (50.6)	867 (51.7)
T3	20 (9.3)	29 (13.7)	12 (8.2)	14 (9.3)	32 (8.8)	43 (11.9)	228 (13.6)	228 (13.6)
T4	6 (2.8)	4 (1.9)	4 (2.7)	1 (0.7)	10 (2.8)	5 (1.4)	58 (3.5)	59 (3.5)
Histological grade, No. (%)								
1	14 (6.5)	16 (7.6)	14 (9.5)	22 (14.7)	28 (7.7)	38 (10.5)	145 (8.6)	140 (8.3)
2	85 (39.4)	85 (40.3)	53 (36.1)	61 (40.7)	138 (38.0)	146 (40.4)	731 (43.5)	708 (42.2)
3	115 (53.2)	108 (51.2)	79 (53.7)	66 (44.0)	194 (53.4)	174 (48.2)	765 (45.5)	787 (46.9)
ER status, No. (%)								
ER positive	160 (74.1)	170 (80.6)	118 (80.3)	119 (79.3)	278 (76.6)	289 (80.1)	1319 (78.5)	1316 (78.4)
ER negative	55 (25.5)	38 (18.0)	29 (19.7)	31 (20.7)	84 (23.1)	69 (19.1)	349 (20.8)	355 (21.2)
ER unknown	1 (0.50)	3 (1.4)	0 (0.0)	0 (0.0)	1 (0.3)	3 (0.8)	13 (0.8)	7 (0.4)
PR status, No. (%)								
PR positive	71 (32.9)	62 (29.4)	60 (40.8)	56 (37.3)	131 (36.1)	118 (32.7)	725 (43.1)	698 (41.6)
PR negative	37 (17.1)	40 (1908)	26 (17.7)	36 (24.0)	63 (17.4)	76 (21.1)	382 (22.7)	424 (25.3)
PR unknown	107 (49.5)	108 (51.2)	61 (41.5)	58 (38.7)	168 (46.3)	166 (46.0)	571 (34.0)	548 (32.7)
HER2 status, No. (%)								
HER2 positive	27 (12.5)	33 (15.6)	14 (9.5)	13 (8.7)	41 (11.3)	46 (12.7)	192 (11.4)	223 (13.3)
HER2 negative	60 (27.8)	54 (25.6)	52 (35.4)	40 (26.7)	112 (30.9)	94 (26.0)	648 (38.5)	603 (35.9)
HER2 unknown/not measured	129 (59.7)	124 (58.8)	81 (55.1)	97 (64.7)	210 (57.9)	221 (61.2)	831 (49.5)	841 (50.1)
Menopausal status, No. (%)								
Pre-menopausal	94 (43.5)	94 (44.5)	69 (46.9)	60 (40.0)	163 (44.9)	154 (42.7)	751 (44.7)	753 (44.9)
≤ 5 years since menopause	32 (14.8)	38 (18.0)	19 (12.9)	22 (14.7)	51 (14.0)	60 (16.6)	247 (14.7)	243 (14.5)
> 5 years since menopause	74 (34.3)	57 (27.0)	45 (30.6)	56 (37.3)	119 (32.8)	113 (31.3)	519 (30.9)	522 (31.1)
Menopausal status unknown	16 (7.4)	22 (10.4)	14 (9.5)	12 (8.0)	30 (8.3)	34 (9.4)	164 (9.8)	160 (9.5)
Planned systemic therapy, No. (%)								
Endocrine therapy alone	18 (8.3)	13 (6.2)	6 (4.1)	7 (4.7)	24 (6.6)	20 (5.5)	76 (4.5)	76 (4.5)
Chemotherapy alone	56 (25.9)	42 (19.9)	25 (17.0)	30 (20.0)	81 (22.3)	72 (19.9)	361 (21.3)	358 (21.3)
Endocrine therapy plus chemotherapy	142 (65.7)	156 (73.9)	116 (78.9)	113 (75.3)	258 (71.1)	269 (74.5)	1244 (74.0)	1244 (74.1)
Type of chemotherapy, No. (%)								
Anthracyclins	194 (89.8)	193 (91.5)	134 (91.2)	139 (92.7)	328 (90.4)	332 (92.0)	1568 (97.7)	1564 (97.6)
Taxanes	35 (16.2)	29 (13.7)	14 (9.5)	15 (10.0)	49 (13.5)	44 (12.2)	390 (24.3)	385 (24.0)
Timing of chemotherapy								
Neo-adjuvant	7 (3.2)	5 (2.4)	2 (1.4)	3 (2.0)	9 (2.5)	8 (2.2)	103 (6.5)	104 (6.5)
Postoperative	209 (96.8)	206 (97.6)	145 (98.6)	147 (98.0)	354 (97.5)	353 (97.8)	1502 (93.6)	1498 (93.5)
Statin use, No. (%)	9 (4.2)	9 (4.3)	7 (4.8)	8 (5.3)	16 (4.4)	17 (4.7)	97 (5.8)	100 (6.0)
Type of first disease-free survival event, No. (%)								
Loco-regional recurrence	16 (7.4)	10 (4.7)	12 (8.2)	5 (3.3)	28 (7.7)	15 (4.2)	79 (4.7)	78 (4.7)
Distant recurrence	41 (19)	44 (20.9)	21 (14.3)	32 (21.3)	62 (17.1)	76 (21.1)	332 (19.8)	341 (20.3)
Distant and loco-regional recurrence	5 (2.3)	2 (0.9)	0 (0)	1 (0.7)	5 (1.4)	3 (0.8)	18 (1.1)	21 (1.3)
Death without prior recurrence	6 (2.8)	9 (4.3)	1 (0.7)	3 (2)	7 (1.9)	12 (3.3)	44 (2.6)	53 (3.2)
First distant recurrence is nonskeletal, No. (%)	31 (14.4)	18 (8.5)	10 (6.8)	17 (11.3)	41 (11.3)	35 (9.7)	194 (11.5)	165 (9.8)
First distant recurrence is skeletal and other, No. (%)	15 (6.9)	28 (13.3)	11 (7.5)	16 (10.7)	26 (7.2)	44 (12.2)	156 (9.3)	197 (11.7)
First distant recurrence is skeletal only, No. (%)*	7 (3.2)	22 (10.4)	11 (7.5)	9 (6)	18 (5)	31 (8.6)	97 (5.8)	140 (8.3)

* This group is a subset of those classified as skeletal and other, where skeletal recurrences were the only first distant recurrences.

### Association of CAPG and GIPC1 With Skeletal Metastasis

#### Training Set

**Figure 3.**
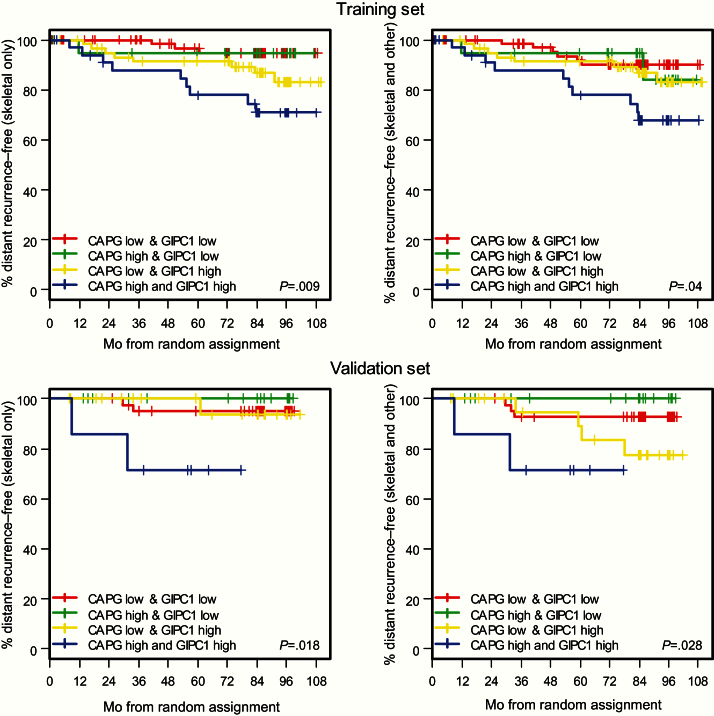
Control arm of the AZURE trial population. Kaplan-Meier estimates of the survival function showing association between protein expression and time to first distant recurrences (DRs). **Upper left**) Training set; first DR is skeletal only. **Upper right**) Training set; first DR includes skeletal as well as other distant site(s). **Lower left**) Validation set; first DR is skeletal only. **Lower right**) Validation set; first DR includes skeletal as well as other distant site(s). High indicates a tissue microarray (TMA) score of 3. Low indicates TMA score of 1 or 2. *P* values refer to the two-sided log-rank test. CAPG = macrophage-capping protein; GIPC1 = PDZ domain–containing protein GIPC1.

Analysis of the control arm data suggested that patients with high CAPG and GIPC1 scores (CAPG^hi^/GIPC1^hi^) had an increased risk of developing distant skeletal events. Figure 3 shows that when either CAPG or GIPC1 is high the risk of developing a distant skeletal event is greater than when both scores are low and that patients who are CAPG^hi^/GIPC1^hi^ have the greatest risk. This is true whether the first distant recurrence event recorded was in bone alone or skeletal plus another distant site (skeletal and other). These data led to analyses considering the potential biomarkers individually and also as a simple bivariate score, where the number of high protein expressions is summed on a scale of 0 to 2, (ie, 0 = both low; 1 = one low, one high; 2 = both high). Results from these analyses confirm that in the control arm CAPG and GIPC1 independently have prognostic potential as biomarkers for development of bone metastasis, CAPG showing a weak association and GIPC1 a stronger association with bone-only metastasis (Supplementary Tables 4 and 5, available online). However, Figure 3 and Table 3 show that this prognostic potential for bone-only metastasis as first distant event is strongly enhanced when both CAPG and GIPC1 are high, treated as a simple bivariate score (HR = 3.50, 95% CI = 1.48 to 8.32, *P* = .004), and this also extends to patients where both skeletal and other distant recurrences are recorded as first event. Such associations were not observed in distant events not involving the skeleton (Table 3).

**Table 3.** Univariate associations of distant recurrence outcomes with biomarker expression in control and ZA arms*

Site of metastasis	Training set				Validation set				Combined sets			
	Control		Zoledronate		Control		Zoledronate		Control		Zoledronate	
	HR (95%CI)	n/N	HR (95% CI)	n/N	HR (95% CI)	n/N	HR (95% CI)	n/N	HR (95% CI)	n/N	HR (95% CI)	n/N
Skeletal only	3.50 (1.48 to 8.32)	21/191	1.28 (0.14 to 11.49)	5/187	10.56 (1.72 to 64.91)	6/96	-†	9/97	4.54 (2.11 to 9.78)	27/287	0.48 (0.06 to 3.63)	14/284
*P*		.004		.823		.011				<.001		.473
Skeletal and other	2.94 (1.34 to 6.49)	26/191	0.58 (0.073 to 4.54)	10/187	5.62 (1.11 to 28.37)	10/96	-†	9/97	3.35 (1.67 to 6.70)	36/287	0.34 (0.05 to 2.57)	19/284
*P*		.007		.599		.037				.001		.297
Nonskeletal	0.64 (0.15 to 2.78)	17/191	0.90 (0.31 to 2.60)	27/187	1.87 (0.23 to 15.02)	10/96	1.29 (0.16 to 10.51)	8/97	0.82 (0.25 to 2.73)	27/287	1.01 (0.39 to 2.60)	35/284
*P*		.548		.842		.554		.463		.747		.985

* Estimates are from Cox proportional hazards regressions for bivariate score in training set, validation set, and the combined sets (BiScore both high vs not). Comparisons shown to be statistically significant are also significant in analyses adjusting for the effect of systemic therapy plan, estrogen receptor status, and lymph node involvement. Skeletal and other (first distant recurrence event reported includes skeletal and other site[s] of metastasis); skeletal only (first distant recurrence event only skeletal—this group is a subset of those classified as skeletal and other); nonskeletal (first distant recurrence event does not include skeletal recurrence). n = number of events; N = number at risk.

† No events in high group and hence Cox proportional hazards model is inestimable.

Importantly, statistically significant associations of a high bivariate score with events involving the skeleton were not seen in the corresponding group of patients who received zoledronate (Table 3), indicating a potential predictive effect for treatment response (eg, for skeletal only, HR = 1.28, 95% CI = 0.14 to 11.49, *P* = .823).

#### Validation Set

It was prespecified that for the primary analysis this second set would independently validate the results observed in the training set if the *P* value for the bivariate score was less than .05 for the Cox proportional hazards regression approach, described in the Methods section. As shown in Table 3, these analyses for skeletal events only (*P* = .011) and skeletal and other events (*P* = .037) did indeed independently validate the bivariate score as a prognostic biomarker for bone metastasis (further data shown in Supplementary Tables 4 and 5, available online).

#### Combination of Training and Validation Sets

**Figure 4.**
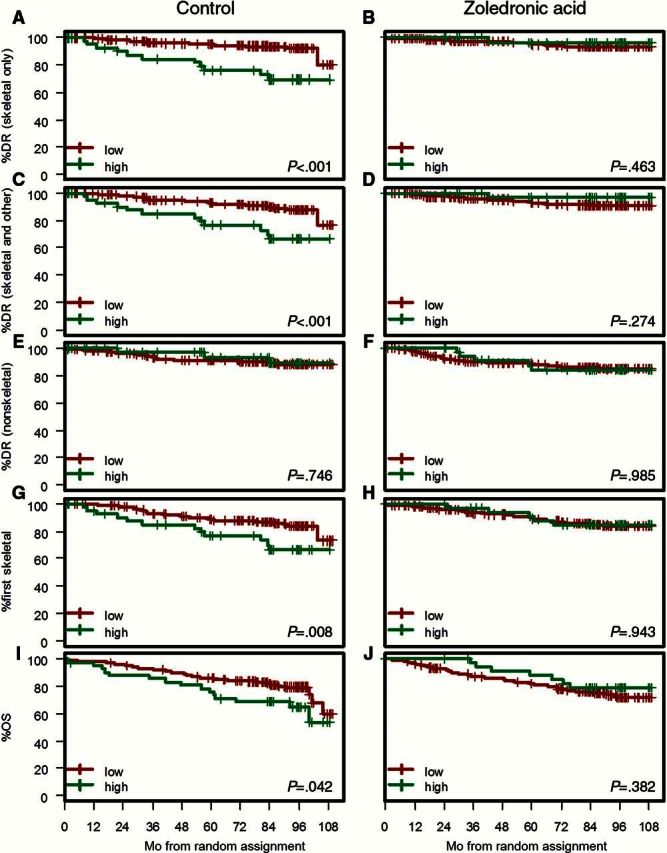
Combined sets. Kaplan-Meier estimates of the survival function for time to distant recurrence and overall survival for control and zoledronate arms. High signifies both macrophage-capping protein (CAPG) and PDZ domain–containing protein GIPC1 (GIPC1) high; low signifies other combinations where not both CAPG and GIPC1 are high. **A and B**) Skeletal only; first distant recurrence only skeletal. **C and D**) Skeletal and other; first report of distant recurrence included skeletal and other site(s) of metastasis. **E and F**) Nonskeletal; first distant recurrence does not contain any skeletal component. **G and H**) First skeletal recurrence irrespective of timing and sites of local and nonskeletal recurrences. **I and J**) Overall survival. *P* values refer to the two-sided log-rank test. DR = distant recurrence; OS = overall survival.

We have carried out further analyses using the greater power obtained by combining the training and validation sets, which correspond to 571 patients scored for both proteins (Table 3). For the control arm, Figure 4 demonstrates the increased power delivered by this large combined dataset, confirming that the composite biomarker CAPG^hi^/GIPC1^hi^ is a highly statistically significant prognostic biomarker for distant recurrence events involving the skeleton. Notably, even with the increased power, there was no statistically significant association of the markers with nonskeletal metastases in either control or zoledronate arms (Table 3). The combined set also confirms the advantage of the combined bivariate score over either CAPG or GIPC1 individually, as demonstrated by the increased hazard ratio values; eg, for skeletal only events, the hazard ratio was 4.54 (95% CI = 2.11 to 9.78, *P* < .001) in the bivariate score (Table 3), compared with 2.92 (95% CI = 1.51 to 5.65, *P* = .001) for GIPC1 and 2.31 (95% CI = 1.14 to 4.69, *P* = .020) for CAPG (Supplementary Tables 4 and 5, available online).

We also considered an alternative cutpoint, ie high vs low between scores of 1 and 2, rather than between scores 2 and 3. This led to very similar results in terms of direction of the effects, and statistical significance and the corresponding version of Figure 4 for this alternative cutpoint is shown in Supplementary Figure 3 (available online).

Supplementary Tables 6 and 7 and Supplementary Figure 1 (available online) also show that the composite biomarker is similarly prognostic for distant recurrence events involving the skeleton when divided into pre/perimenopausal and postmenopausal patient groups.

#### Prediction of Treatment Benefit

**Figure 5.**
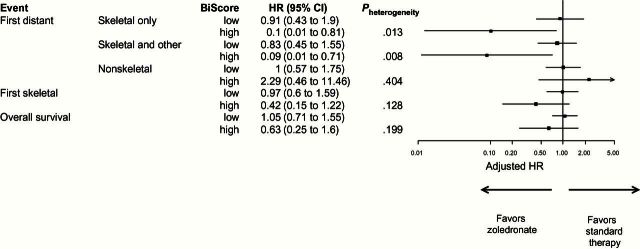
Forest plot showing effect of zoledronate in subgroups defined by the bivariate score composed of macrophage-capping protein and PDZ domain–containing protein GIPC1 in relationship to time to distant recurrence events and overall survival. Skeletal only (first distant recurrence event only skeletal); skeletal and other (first distant recurrence event reported includes skeletal and other site[s] of metastasis); nonskeletal (first distant recurrence event does not include skeletal recurrence); first skeletal (first skeletal event irrespective of whether other distant events have occurred first). All hazard ratios are adjusted for systemic therapy plan, estrogen receptor status, and lymph node involvement. Hazards ratios of less than 1 indicate improvement with zoledronate. CI = confidence interval; HR = hazard ratio.

The effectiveness of zoledronate in reducing the risk of bone metastases in patients that are CAPG^hi^/GIPC1^hi^ is highlighted in Figure 5, while zoledronate has no statistically significant effect in reducing occurrence of nonskeletal metastases. For example, CAPG^hi^/GIPC1^hi^ patients on zoledronate have a 90% reduced hazard of a skeletal-only distant recurrence (adjusted HR = 0.10, 95% CI = 0.01 to 0.81, *P* = .008) as compared with patients on standard therapy; whereas when both CAPG and GIPC1 are not high, patients on zoledronate have a 9% reduced hazard of a skeletal-only distant recurrence (adjusted HR = 0.91, 95% CI = 0.43 to 1.90), with significant heterogeneity in effect between these subgroups (*P* = .013, interaction test). This suggests that CAPG^hi^/GIPC1^hi^ patients attain approximately a 10-fold increase in treatment benefit.

Similar effects are observed for time to skeletal and other distant recurrence (Figure 5), and a similar trend to benefit in CAPG^hi^/GIPC1^hi^ patients is observed in analysis of time to first skeletal event irrespective of other recurrences (*P* = .128). The potential predictive effect of the composite biomarker may also be clearly seen in plots of zoledronate vs control for patients with CAPG^hi^/GIPC1^hi^ and patients who do not have CAPG^hi^/GIPC1^hi^ (Supplementary Figure 4, available online).

### Overall Survival

Based on the combined dataset, CAPG^hi^/GIPC1^hi^ patients in the control arm experienced statistically significantly shorter OS, with a five-year survival of 76.2% (95% CI = 64.4 to 90.3), compared with patients for whom both GIPC1 and CAPG were not high, with a five-year survival of 85.9 (95% CI = 81.7 to 90.4) (HR = 1.81, 95% CI = 1.01 to 3.24, *P* = .045) (Figure 4; Supplementary Table 8, available online). In the zoledronate arm, the corresponding figures for five-year survival were 82.3% (95% CI = 77.6, to 87.3) and 88.2% (95% CI = 78.0 to 99.8) (HR = 0.71, 95% CI = 0.32 to 1.55, *P* = .385), suggesting a treatment benefit of approximately 2.5-fold (comparison of HR values). The corresponding adjusted hazard ratio value (Figure 5) was 0.63 (95% CI = 0.25 to 1.6, *P* = .199).

## Discussion

In this study, we found that a composite biomarker comprising CAPG and GIPC1 in primary breast tumor tissue was not only associated with subsequent development of bone metastasis and reduced survival but was also predictive of the treatment benefits of adjuvant zoledronate. We believe this is the first such validated biomarker to be reported and consequently could be considered for assessment of individual patient risk and selection of patients for adjuvant bisphosphonate treatment. It should be emphasised that even with the increased power of the combined datasets we found no associations between the composite biomarker and development of nonskeletal metastases, further emphasising the specificity of this biomarker for development of bone metastases. This clinical finding also appears to justify our strategy of only taking forward proteomic-derived candidate biomarker proteins upregulated in BM1 and BM2 cells but not in LM cells.

CAPG is a calcium-sensitive, actin-binding protein that plays a role in regulating cytoplasmic and nuclear structures, reported to modify cell migration and invasion (19). High expression of CAPG has been associated with progression and/or metastasis of a range of tumors (20–24) and has been demonstrated in breast cancer cells with increased metastatic potential (6). A recent study has demonstrated that CAPG inhibition reduces breast cancer metastasis in a murine model (25). GIPC1 is a cytoplasmic protein that also localizes to the peripheral membrane, acting as an adaptor protein linking receptor interactions to intracellular signaling pathways, including cell cycle regulation, and expression has been associated with some cancers (26). Overexpression of GIPC1 has been associated with breast tumors (27), and silencing of GIPC1 in MDA-MB-231 breast cancer cells leads to increased apoptotic death, G2 cell cycle arrest, modified cell adhesion, and migration (28). Key proteins of breast cancer progression (including the Akt/Mdm2/p53 axis and IGF-1) are downstream of GIPC1 signaling (10). To date, neither CAPG nor GIPC1 appear to have been studied specifically in the context of breast cancer bone metastasis.

Bone metastatic variants of human breast cancer cell lines, principally MDA-MB-231, have been used to identify proteins important in defining breast cancer metastasis, eg, the role of noggin (29). Also, a comparison of primary breast and bone metastatic tissue with an osteotropic MDA-MB-231 cell line showed a high degree of convergence for proteins up- or downregulated (30), thus validating such cell models. However, our study appears to be the first to fully validate candidates from cell lines in patient tissue prospectively collected for such a purpose, associated with high-quality clinical data. Further studies to elucidate the biological mechanisms through which CAPG and GIPC1 are implicated in bone metastasis development and the effects of antibone resorptive agents on these processes are currently underway in our laboratories.

There are several limitations to this study. TMAs were not available from the whole of the AZURE patient cohort, though the numbers available for analysis and the statistical power achieved suggest that this is not a serious limitation. Although our study has clearly demonstrated the value of the composite biomarker CAPG^hi^/GIPC1^hi^ in both univariate and multivariable analyses, it would be possible in future analyses to explore whether the addition of other novel biomarkers could further enhance prognostic and predictive ability.

The poorer OS in CAPG^hi^/GIPC1^hi^ patients is especially striking and is presumably driven by the as-yet unidentified role of these proteins in promoting bone metastasis. However, because of the zoledronate treatment effect observed in the current study (with HR reduction of up to 10-fold for bone metastases and 2.5-fold for death in CAPG^hi^/GIPC1^hi^ patients) and the restoration of these risks to that of the rest of the breast cancer population studied, the composite biomarker CAPG^hi^/GIPC1^hi^ may have an important future role in the selection of patients most likely to benefit from adjuvant antiresorptive treatment and for stratification in further trials, given that zoledronate has a significant toxicity profile, including osteonecrosis of the jaw in a small proportion of patients.

Future study of this composite biomarker if samples from further datasets become available would be useful and could also enable assessment of whether this biomarker benefit was restricted to zoledronate or applies also to other bone-targeted agents such as clodronate or denosumab.

## Funding

This work was supported by Cancer Research UK (grant number: C18605/A 10048) through a Clinician Scientist Fellowship (to JEB). DAC was supported by a UK Medical Research Council Career Development Fellowship (G0802416).

## Supplementary Material

Supplementary Data

## Notes

The authors are solely responsible for the design of the study; the collection, analysis and interpretation of the data; the writing of the manuscript; and the decision to submit the manuscript for publication. All authors read and approved the final article.

The authors are especially grateful to Professor Joan Massagué for kindly providing the bone- and lung-homing variants of MDA-MB-231 cells used in this study. We also particularly thank the AZURE trial patients and investigators, Mr. Mike Shires and the staff of the Sheffield and Leeds Experimental Cancer Medicine Centres for infrastructure support.
