# Supplementary material for: CAPG and GIPC1: Breast Cancer Biomarkers for Bone Metastasis Development and Treatment
Source: J Natl Cancer Inst. 2016 Jan 12;108(4):djv360. doi: 10.1093/jnci/djv360 (PMC4808632; doi:10.1093/jnci/djv360)
Supplement: Supplementary Data [file supp_108_4_djv360__index.html]

Supplementary Data 

# CAPG and GIPC1: Breast Cancer Biomarkers for Bone Metastasis Development and Treatment

## Supplementary Data

Data files

- Supplementary Data - Supplementary Data
