# Supplementary material for: CAPG and GIPC1: Breast Cancer Biomarkers for Bone Metastasis Development and Treatment
Source: J Natl Cancer Inst. 2016 Jan 12;108(4):djv360. doi: 10.1093/jnci/djv360 (PMC4808632; doi:10.1093/jnci/djv360)
Supplement: Supplementary Data [file supp_djv360_Supplementary_Materials.pdf]

SUPPLEMENTARY MATERIALS

CAPG AND GIPC1: BREAST CANCER BIOMARKERS FOR BONE METASTASIS DEVELOPMENT AND TREATMENT

Westbrook JA, Cairns DA, Peng J, Speirs V, Hanby AM, Holen I, Wood SL, Ottewell PD, Marshall H, Banks RE, Selby PJ, Coleman RE, Brown JE.

INVESTIGATORS' CONTRIBUTIONS

Study conception and design and grant funding: J.E. Brown, R.E. Coleman, P. Selby, R. Banks, I. Holen, J.A.Westbrook.

Acquisition, analysis or interpretation of the data: J.E.Brown, J.A.Westbrook, D.A.Cairns, R.E. Coleman, P. Selby, R. Banks, S.L. Wood, J. Peng, V. Speirs, A.M. Hanby, I. Holen, P.D. Ottewell, H. Marshall.

Drafting, revision and approval of the article: J.E. Brown, J.A. Westbrook, R.E.Coleman, D.A. Cairns, S.L. Wood, P. Selby, R. Banks, I. Holen, P.D. Ottewell, H. Marshall, J. Peng, V. Speirs, A.M. Hanby.

MATERIALS AND METHODS – ADDITIONAL INFORMATION

Clinical validation – immunohistochemistry (IHC)

In order to optimize the staining of breast tumour TMAs for validation of CAPG and GIPC1 on the AZURE trial samples, expression of these proteins was initially characterised using a TMA constructed from 364 unselected breast tumours of various grades obtained from breast tumours diagnosed at the Leeds Teaching Hospitals NHS Trust between 1987 and 2005 (ethics approval granted: Leeds [East] REC:06/Q1206/180). This array was termed GBT. Under expert pathology guidance (AMH), the staining and counting methodology was optimized on this TMA using standard methods as described previously[1,2]. Briefly, five micrometre serial sections of TMA were mounted onto Superfrost Plus slides (BDH, Poole, Dorset), heated at 60 °C for 10 mins, dewaxed in xylene, and rehydrated through graded alcohols (4 x 3 mins). Endogenous peroxidases were blocked (3% hydrogen peroxide, 10 min) and antigen retrieval was performed by heating slides in 10 mM sodium citrate buffer, pH 6.0, using a microwave oven at full power for 20 mins. Slides were cooled on ice for 20 mins and then mounted in Sequenza racks, washed in Tris-buffered saline (TBS), and blocked with goat serum (DAKO) diluted in Zymed (1:10) for 20 mins. Primary antibodies were applied (diluted in Zymed) and left to incubate at 4 °C overnight. Slides were washed with TBS and incubated with HRP-conjugated secondary antibodies (DAKO) for 30 mins. Following another wash with TBS, bound antibodies were visualized using diaminobenzidine before counterstaining with haematoxylin, dehydration, and mounting with coverslips. Antibodies: CAPG primary antibody (Sigma HPA019080, rabbit IgG) was 1/200 and 1/400 for the GBT and AZURE TMAs, respectively, while GIPC1 (abcam ab89684, mouse IgG) primary antibody was 1/50 for both TMAs.

A standard IHC approach of semi-quantitative scoring based on operator assessment of weak to intense staining, using a simple scoring system of 1 (weak expression), 2 (intermediate expression), and 3 (strong expression) was developed such that, for each protein, a wide range of cytoplasmic staining intensity was observed across different graded patient TMA samples. This optimised approach was then applied to patient TMAs from the AZURE study, using two independent patient sets, with scoring by trained operators blinded to the clinical data and under the supervision and checking of pathologist AMH. As detailed in the main paper, the scoring was based on staining intensity only and not on percentage of positivity. This was appropriate for these markers, since when staining was present, it was of a similar intensity throughout the tumour cells, rather than patchy or focal in nature, and therefore the number of stained cells was not an issue, just intensity.

IHC scores were submitted via patient identification numbers to the Leeds Clinical Trials Research Unit, which holds the clinical database for the AZURE study for alignment to the AZURE clinical data and performance of statistical analyses (DAC).

Cell culture and protein extraction

Bone (“BM1”, “BM2”) and lung (“LM”) metastatic variants of the human breast cancer cell line MDA-MB-231, specifically clones 1833, 2287, and 4175, respectively, were supplied by Professor J Massagué (Memorial Sloan Kettering Cancer Center, New York, USA). These metastatic variants have been described in detail[3,4] and have the ability to home to bone (BM1, BM2) or to lung (LM) when administered by intracardiac-injection into immunodeficient mice. The parental cell line, MDA-MB-231, was used as a control (“PCC”), along with a GFP-labeled variant (“GPCC”) as a control for the LM cell line which expressed GFP. To account for variability in the cell system studied, for each cell type, four aliquots of a frozen stock were revived and cultured independently in T150 flasks using Dulbecco’s Modified Eagle Medium, containing D-glucose (25 mM) and sodium pyruvate (1 mM) supplemented with 2 mM L-glutamine and 10% v/v foetal calf serum at 37 °C in 5% CO_2. Confluent cells were harvested, within 10 doublings by disposal of the culture media. Cell-harvest involved three washes with PBS and followed by one wash with 0.35M sucrose. Cells were lysed directly in DTT-free difference gel electrophoresis (DIGE) buffer (7M urea, 2M thiourea, 4% w/v CHAPS, and protease inhibitor cocktail). A 1.0 mL aliquot of DIGE

lysis buffer was distributed across the surface of the cell monolayer and incubated at room temperature for 5 minutes before the cells were removed from the flask surface using a rubber-tipped cell scraper. The lysate was passed through a needle (0.5 x 16 mm) 8-10 times to disrupt genomic material prior to centrifugation in a refrigerated benchtop minifuge (Sigma 1-15K, 13Krpm, 10 minutes, 15 °C). The supernatant was retrieved and stored at -20 °C until needed. Protein content was assessed using a urea-compatible modified version[5] of the dye-binding protein assay method originally described by Bradford[6].

Proteomics

Protein labeling

Samples were diluted to a working concentration of 1 mg/mL with DTT-free DIGE lysis buffer and the pH of 52 µL protein solution per sample was adjusted to pH 8.5 with 2.6 µL 1M Tris-HCl (pH 8.5) prior to CyDye labeling. For 2D-DIGE, a pooled internal standard was prepared (with built-in excess) by mixing equal amounts (67.5 µg) of protein from each of the four extracts per cell line (PCC, GPCC, BM1, BM2). One replicate from the LM cell type was of low yield and was not used to contribute to the internal standard. Consequently, 90 µg from each of the three remaining LM replicates was contributed to the internal standard. Five nanomoles of Cy3 dye (1:5 diluted in dry DMF) was added to the internal standard and labeling was effected by incubating the sample on ice, in the dark, for 30 minutes after which the reaction was stopped using 10 mM lysine. The quenched labeled internal standard was incubated a further 10 minutes on ice in the dark before an equal volume of DTT-containing DIGE buffer was added (7M urea, 2M thiourea, 4% w/v CHAPS, 2% w/v DTT, 1.6% v/v Pharmalyte pH 3-10). For labeling of individual cell extracts, an aliquot of 52.5 µg protein was labeled with 5 nmoles of Cy5 dye and dye was incorporated in the same manner described for Cy3. For each 2D gel used, an equal amount of Cy3-labelled internal standard and Cy5-labelled sample was mixed together (*i.e.* 50 µg per CyDye-labeled sample rendering a total of 100 µg protein per 2D gel) except for the low-yield LM sample – the Cy5 labeled component only being used for this sample. Samples were randomized prior to CyDye labeling and the randomized sequence was maintained throughout the subsequent 2-DE steps.

Two dimensional gel electrophoresis (2-DE)

2-DE was carried out in three phases: (1) isoelectric focusing, (2) equilibration of IPG strips, (3) gel electrophoresis.

(1) Isoelectric focusing (IEF)

Equal amounts of protein (50 µg) of Cy3- and Cy5-labelled sample were mixed and brought to a final volume of 450 µL with rehydration buffer (7M urea, 2M thiourea, 4% w/v CHAPS, 1% w/v DTT, 0.8% v/v Pharmalyte pH 3-10, trace amount of bromophenol blue). The dye-labeled protein mix was applied along the length of a 24 cm pH 4-7 immobilized pH gradient (IPG) strip in an immobiline drystip reswelling tray (GE healthcare) and covered with silicon oil. Protein uptake was facilitated by allowing the IPGs to rehydrate overnight at room temperature. Following IPG rehydration, proteins were separated according to their isoelectric points (pI) in an ETTAN IPGPhor3 (GE Healthcare) using a series of voltages but not exceeding a current of 50 µA maximum per IPG strip: gradient program: 300 V for 3 hrs; gradient from 300V to 3500 V over 5 hrs; hold at 3500 V for 18 hrs; gradient from 3500 to 8000 V over 10 mins and hold at 8000 V for 1 hr.

(2) Equilibration of IPG strips

Following IEF, proteins were reduced using DTT (0.1M) for 15 mins in equilibration solution (6M urea, 30% v/v glycerol, 2% w/v SDS in 0.05M Tris-HCl pH 6.8) and then this solution discarded and the protein alkylated by incubation for a further 15 minutes in equilibration solution with addition of 0.48M IAA. alkylated with IAA (0.48M) for 15 mins in equilibration solution.

(3) Polyacrylamide gel electrophoresis

Following equilibration, IPG strips were placed on top of 10% polyacrylamide gels (255 x 196 x 1 mm) and sealed in place with molten agarose (1.5% w/v). Gels were cast between low fluorescence glass panes (GE Healthcare) using an automated mixer (a2DEoptimizer, NextGen Sciences Inc.): acrylamide:bisacrylamide 37.5:1 (40% T, 2.65% C), Tris 2.25M (pH 8.8), SDS 0.6% w/v, TEMED 0.9% v/v, ammonium persulfate 1% w/v. Proteins were separated

according to molecular weight using an ETTAN DALT*twelve* system at 20 mA/gel, 15 °C, until the migration marker (bromophenol blue) reached the bottom of the gels. Running buffer, 10X, (0.25M Tris, pH 8.3, 1.92M glycine, 1% w/v SDS) was diluted to 2X and 1X for upper and lower chambers, respectively.

Gel image analysis

Immediately following electrophoresis, gels were scanned for image capture using a Typhoon 9400 Variable Mode Imager (GE Healthcare). Gels were kept in the dark, foil-covered whilst being queued for scanning so as to minimize effects of ambient light that could reduce fluorescent intensity. Photomultiplier tube voltages were carefully tested to capture images with signal intensities of ~70,000-90,000 counts (out of 100,000 maximum) per Cy3 and Cy5 channel. As soon as a gel had been scanned, it was carefully transferred to a glass dish containing fixing solution (50% v/v methanol, 10% v/v acetic acid, 40% v/v ultra-pure water ($\leq 18.2\times10^6$ ohm/cm)). Following a minimum of overnight fixation, the gels were silver stained using a kit (ProteoSilver™ Silver Stain Kit, Sigma) following the manufacturer's instructions but modified to use 300 mL solution per step. Silver stained gels were scanned using an ImageScanner III (GE Healthcare) and stored carefully at 4 °C in gel bags until required for mass spectrometry.

DIGE data analysis

All major analytical steps were undertaken using Microsoft Excel and the R software environment[7] for statistical computing (v.3.1 run in RStudio v.0.98.507)[8]. Quantitative data for 1,292 DIGE spots matched across all n=20 gels in the dataset (n=4 gels per cell type) was exported as normalised volumes, NV. Initially, the data was visualised using the scatterplotMatrix function in R which indicated that NVs were non-normally distributed suggesting that the use of non-parametric statistical tests would be appropriate. Options to normalise the data, *e.g.* log-transformation, were not explored since it has been reported that, *e.g.*, the Wilcoxon-Mann-Whitney test retains 95.5% of the power the *t*-test would have to analyse the data even for moderate size samples[9]. The scatterplot matrix also demonstrated strong correlation between the PCC and GPCC, and BM1 and BM2, cell types which was supported by a principal components analysis and dendrogram (Figures S5).

The preliminary survey of the NVs indicated relevant and appropriate clustering of samples by cell type within the data, and the potential for underlying differential protein expression driving observed differences between cell types. Consequently a comprehensive analysis of the NV data was undertaken using a custom script created and run in R that calculated descriptive statistics (the five-number summary along with mean NV per cell type and standard deviation), pairwise comparisons between all cell types (reporting the ratio of NV and p-value (Wilcoxon-Mann-Whitney)), Kruskal-Wallis one-way analysis of variance by ranks (KW) p-values, and false discovery rate (FDR) using the q-value method[10]. The KW test is equivalent to one-way ANOVA and is a powerful non-parametric alternative to the *F*-test which can be used to test whether *k* independent groups are from the same population without all of the restrictive assumptions required by the parametric test. This analysis was undertaken using the kruskal.test function in R. The FDR is a method for assessing significance when a number of significance tests are performed simultaneously. In this case these significance tests are comparisons of NV between different classes for 1,292 DIGE spots. The FDR controls the expected proportion of incorrectly rejected null hypotheses (type I errors) in the set of tests which are called significant. An early method of controlling the FDR was based on the Simes inequality fixing a level at which to control the FDR[11]. The *q*-value[10] is a method of estimating the FDR analogous to the *p*-value in classical significance testing. The *q*-value of an individual hypothesis test is the minimum FDR at which the test can be called significant. These figures are calculated for each significance test applied here. They are chosen over other methods as they are convenient and are valid in cases where tests cannot be said to be independent. The values were obtained using the library (qvalue) package in R.

Gel spot excision

Following quantitative analyses, gel spots featuring significant changes in expression (BM / PCC is > 1.5 fold for spots increased in BM or BM / PCC is < 0.66 for spots decreased in BM vs. PCC, with *p*-value < 0.05) were visually checked on DIGE gel images prior to inclusion on a 'pick list'. Each gel spot was assigned a unique number and manually matched to the appropriate location on the silver stained DIGE gels using the PG240 component of the

SameSpots software package. Using the silver stained DIGE gels facilitated accurate localisation of gel spots of interest without recourse to a separately prepared and stained preparative gel thereby significantly reducing the chance of erroneous selection for identification by mass spectrometry. Gel spots were excised manually from silver stained DIGE gels using a 100 µL pipette tip cut to achieve an internal diameter of ~1.5 – 2.0 mm. A gel spot of interest was excised from two or more silver stained gels and these were collected to one 1.5 mL capacity Eppendorf tube to ensure a sufficient amount of material for MS identification. The excised gel spots were covered with a small volume of ultra-pure water, and stored at 4 °C until needed.

Protein identification - Mass spectrometry

Tryptic peptides were generated for mass spectrometric analysis using an in-gel digestion method. Briefly, gel spots were subjected to rounds of dehydration and rehydration using 100% acetonitrile and 100 mM ammonium hydrogen carbonate (“ambic”), respectively, and reduced and alkylated with 10mM DTT and 55 mM IAA, respectively, to render the gel framework accessible to, and suitable for, trypsin and to ensure complete protein reduction and alkylation before proceeding. Proteins were digested overnight in an incubator (37 °C, 5% CO_2) using a trypsin : protein ratio (µg) of 1:40. Peptides were extracted from gel pieces using rounds of dehydration and rehydration with 100% acetonitrile and 50 mM ambic, respectively, with incubations in a sonicating water bath during each step. Peptides were collected by brief centrifugation following each step and supernatants were pooled into an Eppendorf tube. Peptide solution was incubated at -20 °C for 45 minutes and concentrated to a minimal volume (Concentrator Plus, Eppendorf) and stored at -20 °C until needed for mass spectrometry.

HPLC/MS/MS analysis

Nano-LC/MS/MS analysis was performed on a QSTAR XL quadrupole time-of-flight hybrid mass spectrometer (AB Sciex, Warrington, UK) coupled online with an Agilent 1100 Series nano-LC System (Agilent Technologies, Berkshire, UK) through electrospray ionization. The nano-HPLC system was configured to use a trap column (5 mm x 0.3 mm ID, Zorbax 300SB-C18, 5 µm, Agilent Technologies) to desalt and retain tryptic peptides, and a micro-switch to switch the trap column into the flow path of the nano-analytical column (15 cm x 75 µm ID, Zorbax 300SB-C18, 3.5 µm, Agilent Technologies). The mobile phases used were 0.1% v/v formic acid (Eluent A) and ACN, 0.1% v/v formic acid (Eluent B). The tryptic peptides were dissolved in 10 µL 0.1% v/v trifluoroacetic acid and 5 µL was injected onto the trap column at a flow rate of 20 µL/min in eluent A. After 8 min, the trap column was switched to couple with the analytical column running at 200 nL/min with 10% Eluent B. A multistage gradient (10-40% Eluent B over 30 min, 40-90% Eluent B over 5 min, 90% Eluent B for 2 min, 90-10% Eluent B over 2 mins, 10% Eluent B for 15 min) was started immediately after the column switch. In the meantime, the trap column was cleaned by ACN. The eluate from the nano-column was directly ionized by spraying through a nanospray emitter (FS360-20-10-D-20, New Objective, Woburn, MA) in a +2kV electrical field. The mass spectrometer was operated in a data dependent mode: a MS scan from 400-1800 m/z was performed for 1 s; the three most abundant doubly and triply charged ions in the region of m/z 400-1000 and with intensities over 40 counts were selected for MS/MS analysis by collision in a nitrogen gas cell. Each MS/MS analysis was acquired from 80-1800 m/z for 1 s in the Enhance All mode and the precursors were then excluded for 200 s.

MS data analysis

The MS/MS raw data from the LC/MS/MS analysis were processed by Analyst v2.0 and a script plug-in Mascot.dll 1.6b24 (AB Sciex, Warrington, UK) then sent to the local Mascot database search engine (v2.3, Matrix Science, Boston, MA) with the following Mascot searching parameters: Precursor mass tolerance: 0.15Da; Fragment ion mass tolerance: 0.1Da; Enzyme: trypsin allowing 1 missing cleavage site; Fixed modification: carbamidomethyl (C); Variable modification: oxidation (M). Instrument: ESI-QUAD-TOF; Protein sequence database: NCBInr (38032689 sequences, restricted to *Homo sapiens* 276505 sequences). Peptide identifications with scores over threshold ($p<0.05$) were considered significant. The false positive rate was estimated as 0.25% by searching against the decoy database.

Western blotting – verification of protein expression for selected targets

Proteins significantly up-regulated in the BM cell lines only were assessed on the basis of likely relevance to cancer and/or bone metastasis (using published literature). On this basis the following were selected for further study: macrophage-capping protein (CAPG); PDZ domain-containing protein GIPC1 (GIPC1); Transcriptional activator protein Pur-alpha (PURA).

The differential expression of these selected proteins was assessed in cell lysates using Western blotting. This verified increased CAPG and GIPC1 expression (Figure 1, main paper), but increased PURA expression was not observed (data not shown). Protein expression was initially tested using a standard ECL-based detection method, but expression of CAPG and GIPC1 was assessed further using an infra-red (IR) detection system (LiCOR Biosciences). Standard SOPs were used throughout. Briefly, a mini-1D-gel format was utilised using either hand-cast polyacrylamide gels or TGX gels with the mini-Protean system from Bio-Rad. Typically, 5-10 µg cell lysate was mixed with sample loading buffer (Laemmli solution[12]), boiled for 3 mins, and loaded to a mini-gel that was electrophoresed in standard Tris-Glycine buffer at a constant 20mA/gel until the dye front reached the bottom of the gel. Gels were removed from the gel tank and equilibrated in standard transfer buffer (without inclusion of methanol for IR work) for 15 mins before being transferred to a nitrocellulose membrane (GE Healthcare) at a constant 100V for 1 hr. Transfer was checked by assessing the presence and intensity of molecular weight marker transfer and/or using Ponceau Red (Sigma). Immediately following removal of the membrane, the gel was fixed in solution (50:10:40 methanol:acetic acid:water) for 30 mins before being stained overnight with colloidal Coomassie blue (Sigma). The gel was destained and scanned using a densitometer – the images were used for normalisation of immunoprobed blots (see below).

For immunodetection, membranes were blocked for 1hr at room temperature on a shaker with 5% TBST-M (Tris-buffered saline with 1% w/v Tween-20 and 5% w/v Marvel milk powder), for ECL work, or Odyssey blocking buffer (a BSA-based solution from LiCor Biosciences) for IR work. Membranes were incubated in primary antibodies for 1hr in TBST-M or Odyssey blocking buffer. (Antibodies used: CAPG: Abcam ab89511 mouse IgG, 1/5000 – 1/10,000; GIPC1: Abcam ab89684, mouse IgG, 0.5 – 1.5 µg/mL; PURA: Abcam ab77734, mouse IgG, 1.0 – 2.0 µg/mL). Following binding of primary antibody, the membrane was washed 5x5mins in TBS-T (ECL work) or PBS-T (Phosphate-buffered saline with 1% w/v Tween-20, IR work) prior to incubation with species-relevant secondary antibodies diluted in TBST-M or Odyssey blocking buffer for 1 hr. (Secondary antibodies used for ECL work: anti-mouse IgG, HRP-conjugated, Dako, used at 1/100; IR work: #926-68070 IRDye 680RD goat anti-mouse IgG (H + L), LiCor Biosciences, used at 1/5000). Prior to visualisation, membranes were washed thoroughly for 1 hr (3x20mins washes) in TBS-T (ECL) or PBS (IR). For ECL work, membranes were incubated with SuperSignal West Dura chemiluminescent substrate (Thermo Scientific) according to the manufacturer's instructions, and exposed to X-ray film (Kodak). Films were developed using an automated film processing machine, and developed films were scanned using on a densitometer (GE Healthcare). IR blots were imaged using the Odyssey scanner (LiCor Biosciences) with standard conditions (0.67µ resolution, default intensities). Quantitative digitised scan data was extracted and analysed using Excel. Images were normalised against a consistent band of the digitised image of the Coomassie stained gel used for the Western blot transfer. (This method of normalisation can be useful for systems where expression of housekeeping proteins may be perturbed by the underlying biology[13]. Normalised densitometric data from triplicate runs of the immunoprobed samples were tested for significance using the Student's t-test.

Since CAPG and GIPC1 (but not PURA) were verified as having higher expression in BM cell lysates, CAPG and GIPC1 were selected to take forward for clinical validation (see also main paper).

ADDITIONAL BACKGROUND INFORMATION RE: CAPG AND GIPC1 WITHIN BREAST CANCER METASTASIS

CAPG

CAPG is a member of the gelsolin family of proteins and functions to remodel the actin cytoskeleton via capping the plus end of actin filaments in a Ca^{2+} and phospholipid (PIP_2)-dependent manner. CAPG has been identified as an oncogene within a wide variety of cancers including oral-cancers[14], melanoma[15], ovarian cancer[16] and pancreatic cancer[17]. Several studies have implicated CAPG within breast cancer development. CAPG can localize to both the nucleus and the cytoplasm and fluorescence microscopic studies have demonstrated an increased rate of nuclear/cytoplasmic shuttling of CAPG within invasive breast cancer cells[18,19]. Proteomic studies identified increased levels of CAPG protein at the interface zone between breast cancer cells and surrounding tumor microenvironment[20] and recent studies using the intracellular expression of nanobodies to bind and neutralize potential pro-metastatic proteins demonstrated reduced metastatic behavior of breast cancer cells upon targeting of CAPG[21]. Despite these studies implicating CAPG within breast cancer cell motility there have been no studies to date identifying a role of CAPG within metastasis to bone.

GIPC1

GIPC1 is a PDZ-domain containing scaffold protein which functions within the intracellular trafficking of a variety of receptor signaling complexes including: lysophosphatidic-acid receptor LPA_1[22], the TrkA nerve-growth factor receptor[23,24], VEGFR2[25], TGF-beta type III receptors[26] as well as G-protein coupled receptors such as the dopamine-D2 receptor[27] and the lupotropin receptor (hLHR)[28]. In terms of a mechanistic role within cancer progression, the interaction of GIPC1 with the TGF-beta type-III receptor has been demonstrated to be required for epithelial-mesenchymal transition within endothelial cells[26] a process that requires SMAD signaling and the potential functional synergy with the type-I membrane glycoprotein endoglin[29], as well as mediating the anti-migratory effects of the TGF-beta-III receptor[30]. To date there have been no studies linking GIPC to the development of bone metastasis. GIPC1 expression is essential for the growth and survival of MDA-MB-231 cells *in vitro*[31] and the interaction of GIPC1 with MyoGEF has been implicated in the progression of breast cancer[32]. GIPC1 signaling is linked to the several proteins which function with breast cancer tumorigenesis including Akt/MDM2 and p53[31].

SUPPLEMENTARY FIGURES AND TABLES: CLINICAL DATA

Supplementary Figure 1: Combined Training and Validation Sets. Kaplan-Meier estimates for the survival function for time to distant recurrence for control and zoledronate arms according to menopausal status. First distant recurrence includes skeletal and other sites of metastasis. Bivariate score for protein expression (see main paper): 0 = both CAPG and GIPC1 low; 1 = one protein high and the other low; 2 = both CAPG and GIPC1 high. The figure therefore compares patients with both CAPG and GIPC1 high against patients where not both CAPG and GIPC1 are high. P-values refer to the logrank test.

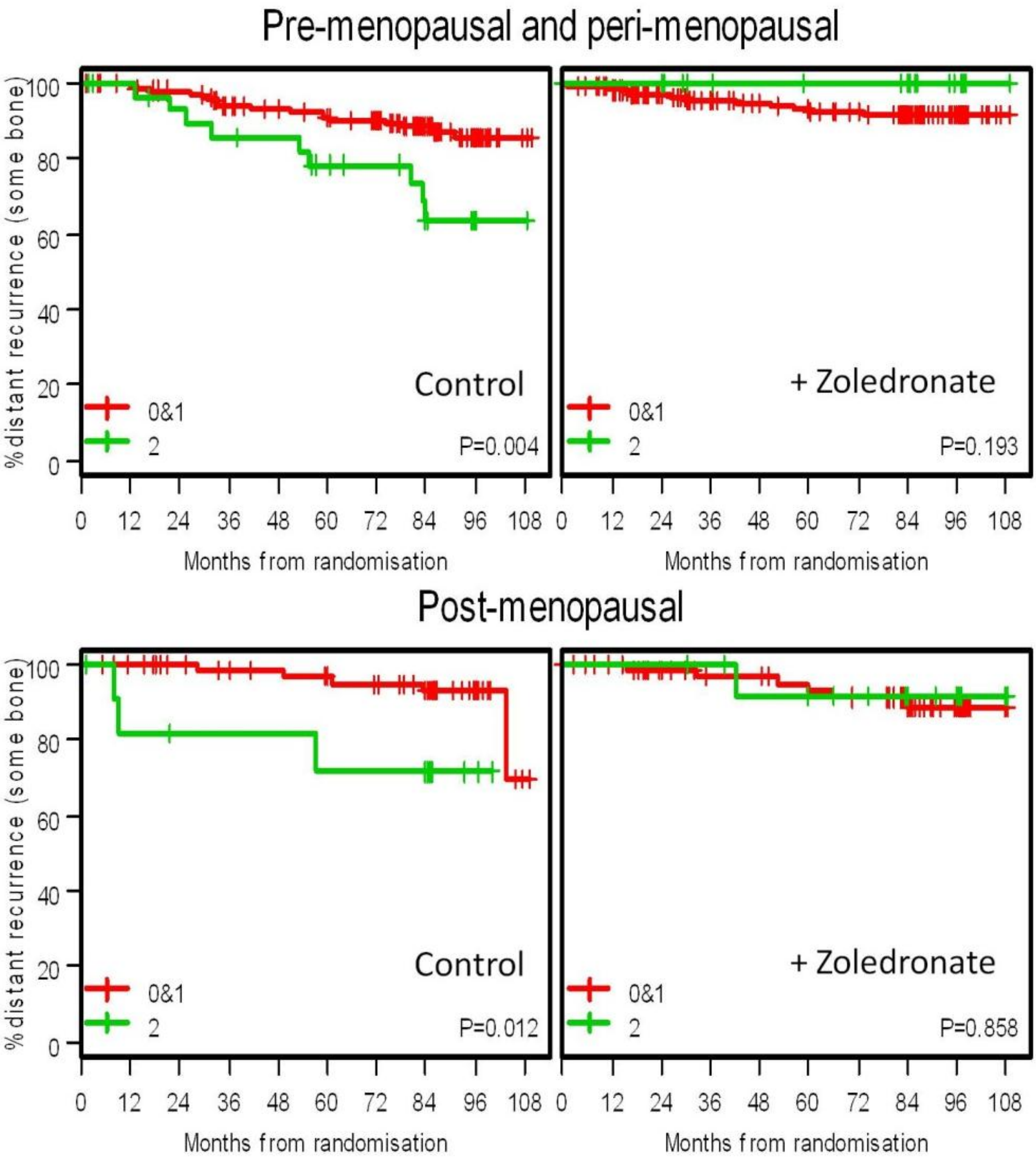

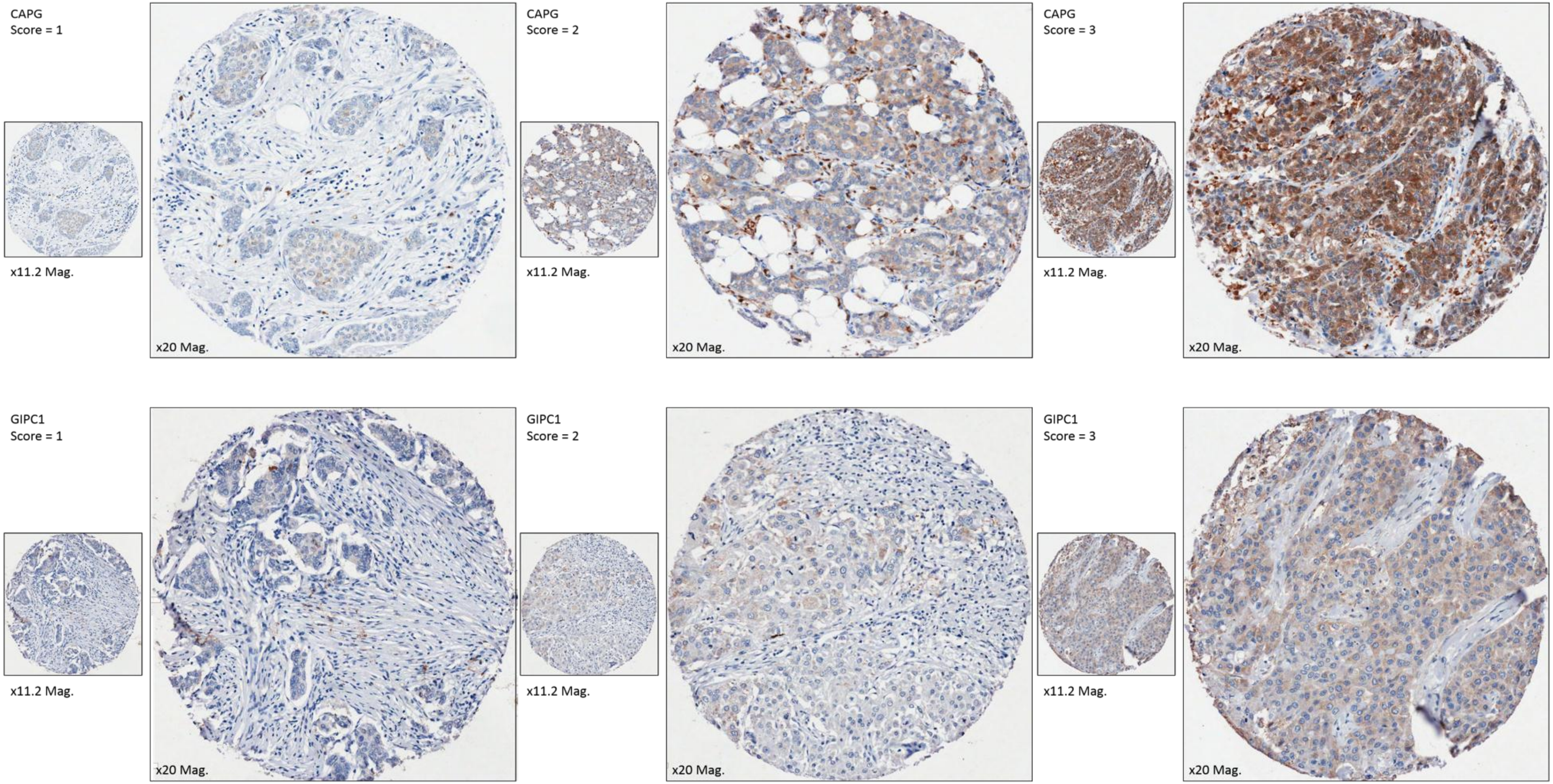

Supplementary Figure 2: Composite of differential protein expression intensity for CAPG (upper panel) and GIPC1 (lower panel) as revealed by IHC and visualised at two magnification levels (x11.2 and x20). As for Figure 2 (main paper), in each case, the antibody localisation has been used in conjunction with DAB (diaminobenzidine, brown), and the scoring is based on the intensity of staining in the cytoplasmic compartment in the tumour cells only.

Supplementary Figure 3: Combined Training and Validation Sets. Kaplan-Meier estimates for the survival function for time to distant recurrence (DR) and overall survival for control and zoledronate arms. High signifies at least one of CAPG or GIPC1 high; Low signifies other combinations. P-values refer to the logrank test.

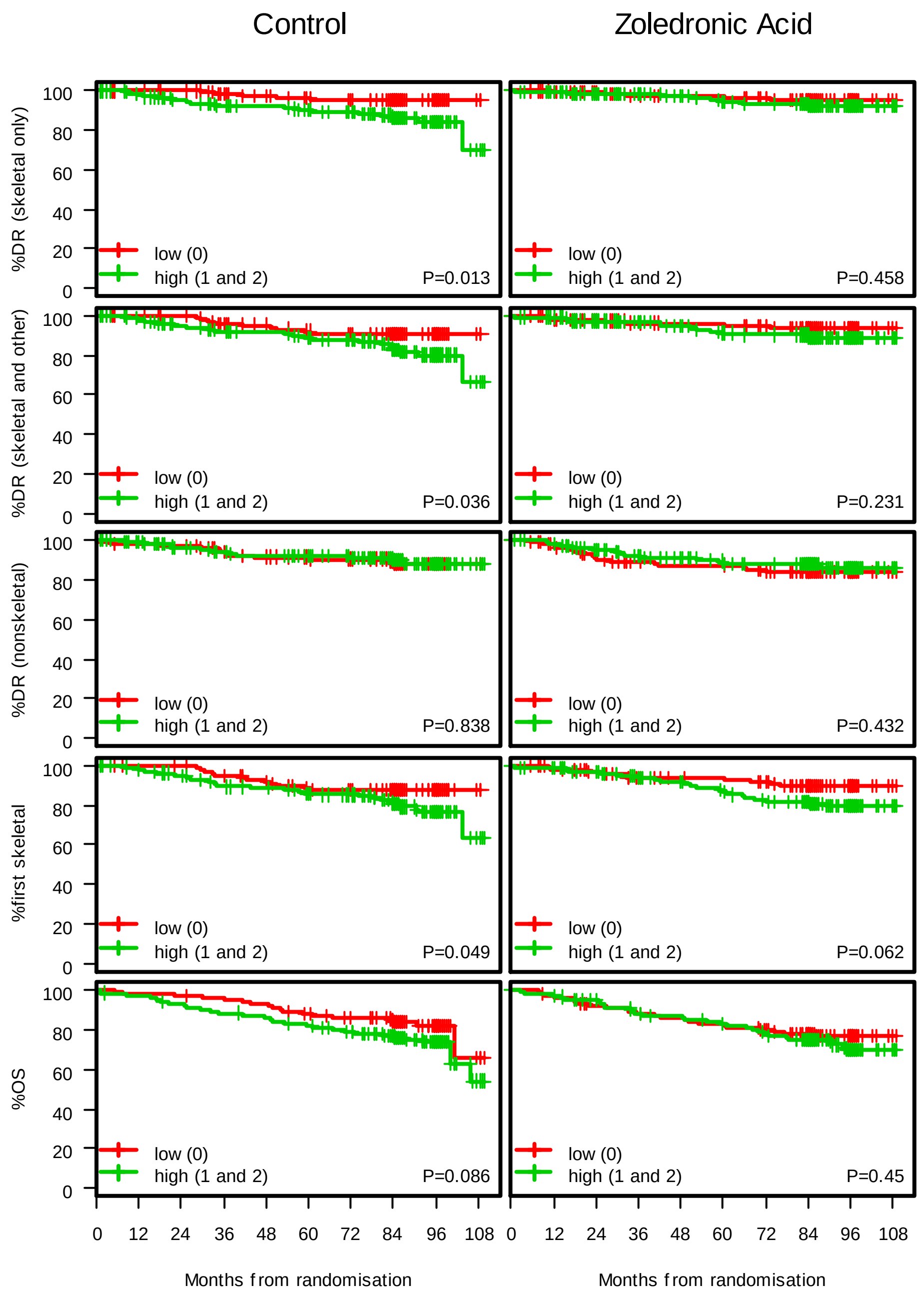

Supplementary Figure 4: Combined Training and Validation Sets. Kaplan-Meier estimates for the survival function for time to distant recurrence (DR) and overall survival by control and zoledronate arms for both CAPG and GIPC1 high and control and zoledronate arms for other combinations where not both CAPG and GIPC1 are high. P-values refer to the logrank test.

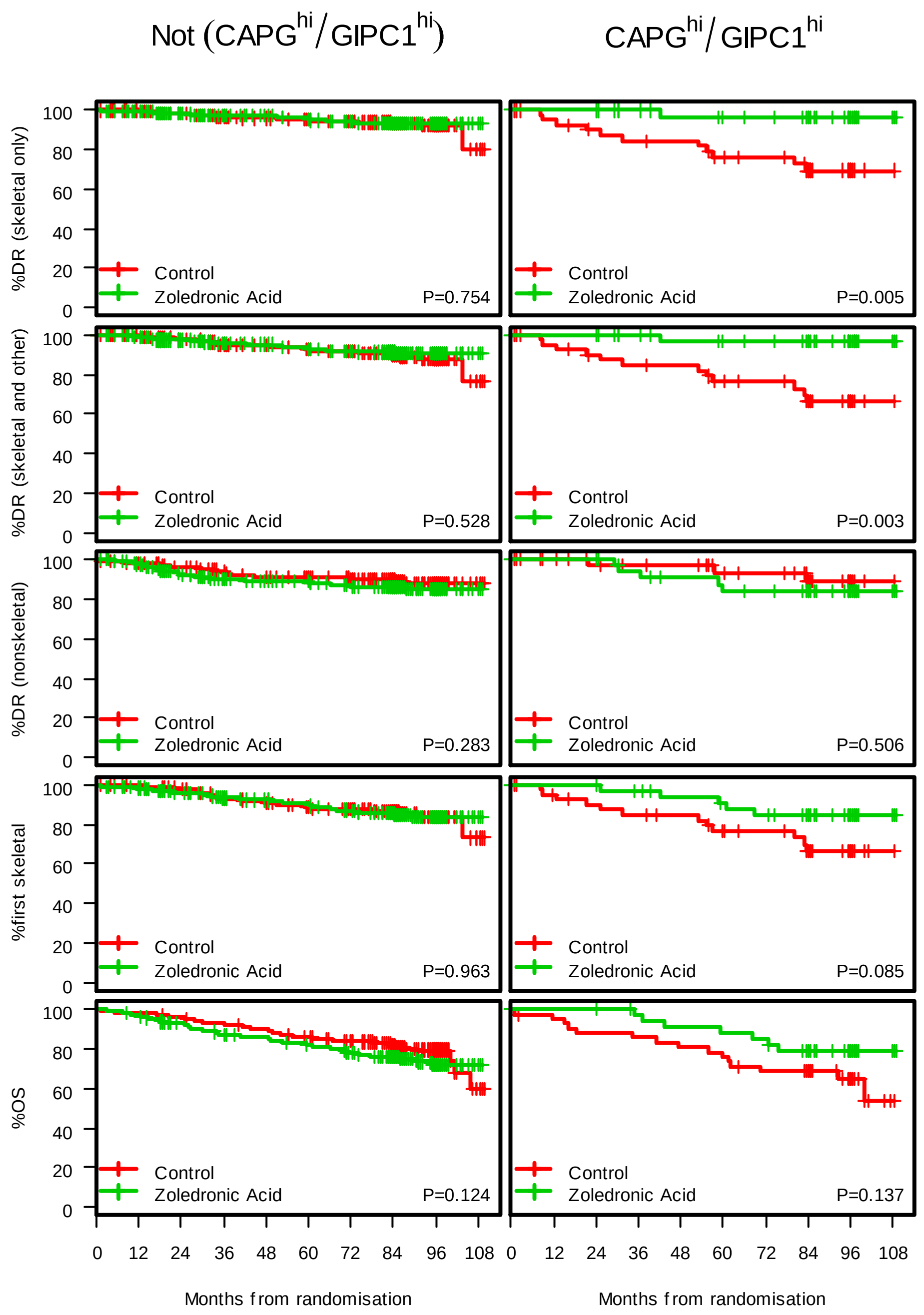

Supplementary Table 1: Associations between CAPG expression and clinical and pathological variables in each arm of the AZURE trial.

	Standard treatment CAPG tumour							Standard treatment + Zoledronic acid CAPG tumour						
**Characteristic**	**1**	**(%)**	**2**	**(%)**	**3**	**(%)**	**p-value**	**1**	**(%)**	**2**	**(%)**	**3**	**(%)**	**p-value**
CAPG														
	67	(18.6)	195	(54)	99	(27.4)		76	(20.9)	196	(54)	91	(25.1)	
Age at Rx (years)														
Median (range)	50	(33, 68)	51	(33, 79)	52	(33, 79)	0.301	49.5	(26, 77)	50	(26, 75)	52	(30, 71)	0.892
Tumor Stage														
T1	15	(10.9)	83	(60.1)	40	(29)	0.099	29	(22.7)	64	(50)	35	(27.3)	0.972
T2	41	(23.4)	86	(49.1)	48	(27.4)		39	(20.2)	108	(56)	46	(23.8)	
T3	10	(23.3)	23	(53.5)	10	(23.3)		6	(18.8)	18	(56.2)	8	(25)	
T4	1	(20)	3	(60)	1	(20)		2	(20)	6	(60)	2	(20)	
Missing	0		0		0			0		0		0		
Histological Grade														
1	2	(5.3)	27	(71.1)	9	(23.7)	0.005	5	(17.9)	16	(57.1)	7	(25)	0.143
2	26	(17.8)	72	(49.3)	48	(32.9)		21	(15.2)	84	(60.9)	33	(23.9)	
3	39	(22.4)	96	(55.2)	39	(22.4)		50	(25.8)	94	(48.5)	50	(25.8)	
Not specified	0	(0)	0	(0)	3	(100)		0		0		0		
Missing	0		0		0			0	(0)	2	(66.7)	1	(33.3)	
ER status														
ER positive	47	(16.3)	159	(55)	83	(28.7)	0.084	54	(19.4)	156	(56.1)	68	(24.5)	0.222
ER negative	18	(26.1)	35	(50.7)	16	(23.2)		21	(25)	40	(47.6)	23	(27.4)	
ER unknown	2	(66.7)	1	(33.3)	0	(0)		1	(100)	0	(0)	0	(0)	
Missing	0		0		0			0		0		0		
Menopausal status														
Pre-menopausal	31	(20.1)	83	(53.9)	40	(26)	0.425	35	(21.5)	89	(54.6)	39	(23.9)	0.816
<= 5 years since menopause	15	(25)	26	(43.3)	19	(31.7)		11	(21.6)	30	(58.8)	10	(19.6)	
> 5 years since menopause	15	(13.3)	66	(58.4)	32	(28.3)		25	(21)	63	(52.9)	31	(26.1)	
status unknown	6	(17.6)	20	(58.8)	8	(23.5)		5	(16.7)	14	(46.7)	11	(36.7)	
Missing	0		0		0			0		0		0		
Systemic therapy plan														
Endocrine therapy alone	0	(0)	14	(70)	6	(30)	0.065	7	(29.2)	14	(58.3)	3	(12.5)	0.271
Chemotherapy alone	19	(26.4)	35	(48.6)	18	(25)		21	(25.9)	38	(46.9)	22	(27.2)	
Endocrine therapy and chemotherapy	48	(17.8)	146	(54.3)	75	(27.9)		48	(18.6)	144	(55.8)	66	(25.6)	
Missing	0		0		0			0		0		0		
Anthracyclines														
Yes	66	(19.9)	176	(53)	90	(27.1)	0.07	66	(20.1)	176	(53.7)	86	(26.2)	0.208
No	1	(3.4)	19	(65.5)	9	(31)		10	(28.6)	20	(57.1)	5	(14.3)	
Missing	0		0		0			0		0		0		
Taxanes														
Yes	10	(22.7)	22	(50)	12	(27.3)	0.702	13	(26.5)	24	(49)	12	(24.5)	0.554
No	57	(18)	173	(54.6)	87	(27.4)		63	(20.1)	172	(54.8)	79	(25.2)	
Missing	0		0		0			0		0		0		

Characteristic	Standard treatment CAPG tumour 1	(%)	2	(%)	3	(%)	p-value	Standard treatment + Zoledronic acid CAPG tumour 1	(%)	2	(%)	3	(%)	p-value
Neo-adjuvant therapy														
Yes	2	(25)	4	(50)	2	(25)	0.904	2	(22.2)	3	(33.3)	4	(44.4)	0.334
No	65	(18.4)	191	(54.1)	97	(27.5)		74	(20.9)	193	(54.5)	87	(24.6)	
Missing	0		0		0			0		0		0		
Statins														
Yes	3	(17.6)	10	(58.8)	4	(23.5)	0.95	7	(43.8)	6	(37.5)	3	(18.8)	0.099
No	64	(18.6)	185	(53.8)	95	(27.6)		69	(19.9)	190	(54.8)	88	(25.4)	
Missing	0		0		0			0		0		0		
Aromatase inhibitor														
Yes	41	(17.3)	128	(54)	68	(28.7)	0.608	32	(16.9)	109	(57.7)	48	(25.4)	0.127
No	26	(21)	67	(54)	31	(25)		44	(25.3)	87	(50)	43	(24.7)	
Missing	0		0		0			0		0		0		
PR status														
Positive	19	(16.1)	69	(58.5)	30	(25.4)	0.207	23	(17.6)	75	(57.3)	33	(25.2)	0.545
Negative	20	(26.3)	40	(52.6)	16	(21.1)		11	(17.5)	35	(55.6)	17	(27)	
Unknown	27	(16.3)	86	(51.8)	53	(31.9)		42	(25)	85	(50.6)	41	(24.4)	
Missing	1	(100)	0	(0)	0	(0)		0	(0)	1	(100)	0	(0)	
HER2 status														
Positive	12	(26.1)	22	(47.8)	12	(26.1)	0.831	15	(36.6)	17	(41.5)	9	(22)	0.086
Negative	14	(14.9)	54	(57.4)	26	(27.7)		18	(16.1)	61	(54.5)	33	(29.5)	
Unknown	2	(16.7)	6	(50)	4	(33.3)		4	(36.4)	6	(54.5)	1	(9.1)	
Not measured	39	(18.8)	112	(53.8)	57	(27.4)		38	(19.7)	109	(56.5)	46	(23.8)	
Missing	0	(0)	1	(100)	0	(0)		1	(16.7)	3	(50)	2	(33.3)	
Lymph node involvement														
0	1	(20)	3	(60)	1	(20)	0.291	0	(0)	2	(100)	0	(0)	0.542
1-3	39	(16)	139	(57.2)	65	(26.7)		53	(21.5)	127	(51.4)	67	(27.1)	
>=4	27	(23.9)	53	(46.9)	33	(29.2)		23	(20.2)	67	(58.8)	24	(21.1)	
Unknown	0		0		0			0		0		0		
Missing	0		0		0			0		0		0		

Supplementary Table 2: Associations between GIPC1 expression and clinical and pathological variables in each arm of the AZURE trial.

	Standard treatment GIPC1 tumour							Standard treatment + Zoledronic acid GIPC1 tumour						
Characteristic	1	(%)	2	(%)	3	(%)	p-value	1	(%)	2	(%)	3	(%)	p-value
GIPC1														
NA	38	(9.9)	196	(51.3)	148	(38.7)		45	(11.3)	207	(51.9)	147	(36.8)	
Age at Rx (years)														
Median (range)	53	(33, 79)	51	(32, 79)	51.5	(33, 76)	0.526	51	(26, 77)	52	(26, 77)	50	(30, 75)	0.796
Tumor Stage														
T1	18	(12.9)	54	(38.6)	68	(48.6)	0.01	12	(9.4)	73	(57.5)	42	(33.1)	0.235
T2	17	(9.3)	110	(60.1)	56	(30.6)		30	(13.7)	101	(46.1)	88	(40.2)	
T3	3	(5.7)	29	(54.7)	21	(39.6)		3	(7.7)	23	(59)	13	(33.3)	
T4	0	(0)	3	(50)	3	(50)		0	(0)	10	(71.4)	4	(28.6)	
Missing	0		0		0			0		0		0		
Histological Grade														
1	3	(10.3)	15	(51.7)	11	(37.9)	0.889	3	(10.3)	16	(55.2)	10	(34.5)	0.988
2	12	(7.9)	81	(53.3)	59	(38.8)		18	(12.2)	77	(52)	53	(35.8)	
3	23	(11.6)	99	(50)	76	(38.4)		24	(11.1)	109	(50.2)	84	(38.7)	
Not specified	0	(0)	1	(33.3)	2	(66.7)		0	(0)	1	(100)	0	(0)	
Missing	0		0		0			0	(0)	4	(100)	0	(0)	
ER status														
ER positive	31	(10.5)	152	(51.7)	111	(37.8)	0.522	34	(11.4)	152	(51)	112	(37.6)	0.932
ER negative	7	(8.2)	41	(48.2)	37	(43.5)		11	(11)	54	(54)	35	(35)	
ER unknown	0	(0)	3	(100)	0	(0)		0	(0)	1	(100)	0	(0)	
Missing	0		0		0			0		0		0		
Menopausal status														
Pre-menopausal	15	(8.7)	89	(51.4)	69	(39.9)	0.462	22	(12.6)	87	(49.7)	66	(37.7)	0.813
<= 5 years since menopause	9	(15.5)	25	(43.1)	24	(41.4)		8	(11.8)	40	(58.8)	20	(29.4)	
> 5 years since menopause	11	(9.4)	67	(57.3)	39	(33.3)		13	(10.4)	64	(51.2)	48	(38.4)	
status unknown	3	(8.8)	15	(44.1)	16	(47.1)		2	(6.5)	16	(51.6)	13	(41.9)	
Missing	0		0		0			0		0		0		
Systemic therapy plan														
Endocrine therapy alone	1	(4.3)	12	(52.2)	10	(43.5)	0.544	1	(4.2)	10	(41.7)	13	(54.2)	0.354
Chemotherapy alone	6	(7.1)	40	(47.6)	38	(45.2)		9	(9.3)	55	(56.7)	33	(34)	
Endocrine therapy and chemotherapy	31	(11.3)	144	(52.4)	100	(36.4)		35	(12.6)	142	(51.1)	101	(36.3)	
Missing	0		0		0			0		0		0		
Anthracyclines														
Yes	34	(9.8)	180	(51.7)	134	(38.5)	0.791	42	(11.5)	192	(52.5)	132	(36.1)	0.573
No	4	(11.8)	16	(47.1)	14	(41.2)		3	(9.1)	15	(45.5)	15	(45.5)	
Missing	0		0		0			0		0		0		
Taxanes														
Yes	5	(10)	27	(54)	18	(36)	0.898	4	(7.1)	32	(57.1)	20	(35.7)	0.515
No	33	(9.9)	169	(50.9)	130	(39.2)		41	(12)	175	(51)	127	(37)	
Missing	0		0		0			0		0		0		
Neo-adjuvant therapy														

Characteristic	Standard treatment GIPC1 tumour 1	(%)	2	(%)	3	(%)	p-value	Standard treatment + Zoledronic acid GIPC1 tumour 1	(%)	2	(%)	3	(%)	p-value
Yes	0	(0)	6	(60)	4	(40)	0.81	0	(0)	10	(83.3)	2	(16.7)	0.121
No	38	(10.2)	190	(51.1)	144	(38.7)		45	(11.6)	197	(50.9)	145	(37.5)	
Missing	0		0		0			0		0		0		
Statins														
Yes	4	(22.2)	9	(50)	5	(27.8)	0.178	0	(0)	13	(68.4)	6	(31.6)	0.201
No	34	(9.3)	187	(51.4)	143	(39.3)		45	(11.8)	194	(51.1)	141	(37.1)	
Missing	0		0		0			0		0		0		
Aromatase inhibitor														
Yes	25	(10.5)	130	(54.9)	82	(34.6)	0.113	20	(10)	109	(54.2)	72	(35.8)	0.552
No	13	(9)	66	(45.5)	66	(45.5)		25	(12.6)	98	(49.5)	75	(37.9)	
Missing	0		0		0			0		0		0		
PR status														
Positive	15	(11.4)	73	(55.3)	44	(33.3)	0.346	17	(12.1)	73	(51.8)	51	(36.2)	0.578
Negative	7	(8.3)	37	(44)	40	(47.6)		10	(12.3)	47	(58)	24	(29.6)	
Unknown	15	(9.1)	86	(52.1)	64	(38.8)		18	(10.2)	87	(49.4)	71	(40.3)	
Missing	1	(100)	0	(0)	0	(0)		0	(0)	0	(0)	1	(100)	
HER2 status														
Positive	4	(7.1)	25	(44.6)	27	(48.2)	0.644	5	(10.4)	24	(50)	19	(39.6)	0.959
Negative	11	(11.7)	51	(54.3)	32	(34)		13	(11.5)	56	(49.6)	44	(38.9)	
Unknown	0	(0)	8	(57.1)	6	(42.9)		1	(7.7)	6	(46.2)	6	(46.2)	
Not measured	23	(10.6)	112	(51.6)	82	(37.8)		26	(11.8)	119	(54.1)	75	(34.1)	
Missing	0	(0)	0	(0)	1	(100)		0	(0)	2	(40)	3	(60)	
Lymph node involvement														
0	1	(25)	1	(25)	2	(50)	0.38	0	(0)	4	(100)	0	(0)	0.056
1-3	23	(8.8)	133	(50.8)	106	(40.5)		35	(13.2)	141	(53.2)	89	(33.6)	
>=4	14	(12.1)	62	(53.4)	40	(34.5)		10	(7.7)	62	(47.7)	58	(44.6)	
Unknown	0		0		0			0		0		0		
Missing	0		0		0			0		0		0		

Supplementary Table 3: Associations between BiScore expression and clinical and pathological variables in each arm of the AZURE trial.

	Standard treatment Bivariate score							Standard treatment + Zoledronic acid Bivariate score						
**Characteristic**	**0**	**(%)**	**1**	**(%)**	**2**	**(%)**	**p-value**	**0**	**(%)**	**1**	**(%)**	**2**	**(%)**	**p-value**
Biscore														
(n)	117	(40.8)	127	(44.3)	43	(15)		123	(43.3)	124	(43.7)	37	(13)	
Age at Rx (years)														
Median (range)	50	(33, 79)	52	(33, 79)	53	(33, 75)	0.868	51	(26, 77)	49.5	(30, 75)	52	(36, 67)	0.383
Tumor Stage														
T1	36	(31.9)	57	(50.4)	20	(17.7)	0.21	43	(45.3)	41	(43.2)	11	(11.6)	0.945
T2	62	(47.3)	53	(40.5)	16	(12.2)		64	(41.6)	67	(43.5)	23	(14.9)	
T3	18	(46.2)	15	(38.5)	6	(15.4)		12	(46.2)	11	(42.3)	3	(11.5)	
T4	1	(25)	2	(50)	1	(25)		4	(44.4)	5	(55.6)	0	(0)	
Missing	0		0		0			0		0		0		
Histological Grade														
1	14	(50)	10	(35.7)	4	(14.3)	0.073	7	(35)	10	(50)	3	(15)	0.871
2	46	(41.8)	44	(40)	20	(18.2)		48	(46.6)	42	(40.8)	13	(12.6)	
3	57	(38.8)	73	(49.7)	17	(11.6)		66	(41.8)	71	(44.9)	21	(13.3)	
Not specified	0	(0)	0	(0)	2	(100)		0		0		0		
Missing	0		0		0			2	(66.7)	1	(33.3)	0	(0)	
ER status														
ER positive	93	(41.2)	99	(43.8)	34	(15)	0.413	90	(43.3)	89	(42.8)	29	(13.9)	0.784
ER negative	21	(36.2)	28	(48.3)	9	(15.5)		32	(42.7)	35	(46.7)	8	(10.7)	
ER unknown	3	(100)	0	(0)	0	(0)		1	(100)	0	(0)	0	(0)	
Missing	0		0		0			0		0		0		
Menopausal status														
Pre-menopausal	52	(40.3)	57	(44.2)	20	(15.5)	0.461	55	(43.7)	58	(46)	13	(10.3)	0.643
<= 5 years since menopause	15	(32.6)	26	(56.5)	5	(10.9)		23	(51.1)	17	(37.8)	5	(11.1)	
> 5 years since menopause	38	(44.2)	36	(41.9)	12	(14)		36	(40)	40	(44.4)	14	(15.6)	
status unknown	12	(46.2)	8	(30.8)	6	(23.1)		9	(39.1)	9	(39.1)	5	(21.7)	
Missing	0		0		0			0		0		0		
Systemic therapy plan														
Endocrine therapy alone	5	(29.4)	9	(52.9)	3	(17.6)	0.68	5	(29.4)	11	(64.7)	1	(5.9)	0.512
Chemotherapy alone	22	(36.7)	27	(45)	11	(18.3)		31	(43.7)	32	(45.1)	8	(11.3)	
Endocrine therapy and chemotherapy	90	(42.9)	91	(43.3)	29	(13.8)		87	(44.4)	81	(41.3)	28	(14.3)	
Missing	0		0		0			0		0		0		
Anthracyclines														
Yes	110	(41.8)	114	(43.3)	39	(14.8)	0.466	113	(43.5)	112	(43.1)	35	(13.5)	0.804
No	7	(29.2)	13	(54.2)	4	(16.7)		10	(41.7)	12	(50)	2	(8.3)	
Missing	0		0		0			0		0		0		
Taxanes														
Yes	16	(44.4)	14	(38.9)	6	(16.7)	0.773	18	(45)	17	(42.5)	5	(12.5)	0.97
No	101	(40.2)	113	(45)	37	(14.7)		105	(43)	107	(43.9)	32	(13.1)	
Missing	0		0		0			0		0		0		

Characteristic	Standard treatment Bivariate score 0	(%)	1	(%)	2	(%)	p-value	Standard treatment + Zoledronic acid Bivariate score 0	(%)	1	(%)	2	(%)	p-value
Neo-adjuvant therapy														
Yes	1	(16.7)	4	(66.7)	1	(16.7)	0.441	3	(42.9)	4	(57.1)	0	(0)	0.866
No	116	(41.3)	123	(43.8)	42	(14.9)		120	(43.3)	120	(43.3)	37	(13.4)	
Missing	0		0		0			0		0		0		
Statins														
Yes	8	(57.1)	5	(35.7)	1	(7.1)	0.486	8	(61.5)	4	(30.8)	1	(7.7)	0.495
No	109	(39.9)	122	(44.7)	42	(15.4)		115	(42.4)	120	(44.3)	36	(13.3)	
Missing	0		0		0			0		0		0		
Aromatase inhibitor														
Yes	80	(44)	77	(42.3)	25	(13.7)	0.321	63	(45.7)	52	(37.7)	23	(16.7)	0.069
No	37	(35.2)	50	(47.6)	18	(17.1)		60	(41.1)	72	(49.3)	14	(9.6)	
Missing	0		0		0			0		0		0		
PR status														
Positive	42	(47.2)	36	(40.4)	11	(12.4)	0.593	43	(42.2)	44	(43.1)	15	(14.7)	0.651
Negative	24	(36.9)	32	(49.2)	9	(13.8)		24	(45.3)	20	(37.7)	9	(17)	
Unknown	50	(37.9)	59	(44.7)	23	(17.4)		56	(43.8)	59	(46.1)	13	(10.2)	
Missing	1	(100)	0	(0)	0	(0)		0	(0)	1	(100)	0	(0)	
HER2 status														
Positive	13	(31.7)	20	(48.8)	8	(19.5)	0.327	16	(45.7)	15	(42.9)	4	(11.4)	0.823
Negative	33	(42.9)	36	(46.8)	8	(10.4)		33	(39.3)	36	(42.9)	15	(17.9)	
Unknown	3	(30)	7	(70)	0	(0)		3	(37.5)	4	(50)	1	(12.5)	
Not measured	68	(43)	63	(39.9)	27	(17.1)		71	(46.4)	65	(42.5)	17	(11.1)	
Missing	0	(0)	1	(100)	0	(0)		0	(0)	4	(100)	0	(0)	
Lymph node involvement														
0	2	(50)	1	(25)	1	(25)	0.769	2	(100)	0	(0)	0	(0)	0.126
1-3	78	(39.2)	91	(45.7)	30	(15.1)		88	(45.6)	77	(39.9)	28	(14.5)	
>=4	37	(44)	35	(41.7)	12	(14.3)		33	(37.1)	47	(52.8)	9	(10.1)	
Unknown	0		0		0			0		0		0		
Missing	0		0		0			0		0		0		

Supplementary Table 4: Associations with biomarker expression in control and ZA arms. (Estimates from Cox proportional hazards regressions for CAPG score in Training Set, Validation Set and the combined sets. Comparisons shown to be significant are also significant in analyses adjusting for the effect of systemic therapy plan, ER status and lymph node involvement (n = number of events; N = number at risk). Skeletal only (first distant recurrence event only skeletal); skeletal and other (first distant recurrence event reported includes skeletal and other site(s) of metastasis); Non-skeletal (first distant recurrence event does not include skeletal).

		**Training Set**						**Validation Set**						**Combined sets**					
		**Control**			**Zoledronate**			**Control**			**Zoledronate**			**Control**			**Zoledronate**		
		**HR (95%CI)**	**n/N**	**p-value**	**HR (95%CI)**	**n/N**	**p-value**	**HR (95%CI)**	**n/N**	**p-value**	**HR (95%CI)**	**n/N**	**p-value**	**HR (95%CI)**	**n/N**	**p-value**	**HR (95%CI)**	**n/N**	**p-value**
**Skeletal only**	**CAPG Score** high vs. not	2.16 (0.93-5.00)	22/211	0.072	0.92 (0.18-4.76)	7/216	0.923	2.30 (0.62-8.60)	9/150	0.215	2.46 (0.72-8.41)	11/147	0.152	2.31 (1.14-4.69)	31/361	0.020	1.45 (0.54-3.87)	18/363	0.457
**Skeletal and other**	**CAPG Score** high vs. not	1.95 (0.92-4.11)	28/211	0.081	0.83 (0.27-2.62)	15/216	0.754	1.01 (0.32-3.13)	16/150	0.992	2.46 (0.72-8.41)	11/147	0.152	1.62 (0.87-2.99)	44/361	0.126	1.29 (0.56-2.96)	26/363	0.555
**Non-skeletal**	**CAPG Score** high vs. not	1.01 (0.36-2.82)	18/211	0.99	0.82 (0.37-1.82)	31/216	0.62	2.13 (0.81-5.59)	17/150	0.833	1.03 (0.22-4.84)	10/147	0/974	1.47 (0.73-2.96)	35/361	0.279	0.93 (0.46-1.91)	41/363	0.85

Supplementary Table 5: Associations with biomarker expression in control and ZA arms. (Estimates from Cox proportional hazards regressions for GIPC1 score in Training Set, Validation Set and the combined sets. Comparisons shown to be significant are also significant in analyses adjusting for the effect of systemic therapy plan, ER status and lymph node involvement (n = number of events; N = number at risk). Skeletal only (first distant recurrence event only skeletal); skeletal and other (first distant recurrence event reported includes skeletal and other site(s) of metastasis); Non-skeletal (first distant recurrence event does not include skeletal).

		Training Set						Validation Set						Combined sets					
		**Control**			**Zoledronate**			**Control**			**Zoledronate**			**Control**			**Zoledronate**		
		**HR (95%CI)**	**n/N**	**p-value**	**HR (95%CI)**	**n/N**	**p-value**	**HR (95%CI)**	**n/N**	**p-value**	**HR (95%CI)**	**n/N**	**p-value**	**HR (95%CI)**	**n/N**	**p-value**	**HR (95%CI)**	**n/N**	**p-value**
**Skeletal only**	**GIPC1 Score** high vs. not	2.40 (1.00-5.80)	24/202	0.051	1.07 (0.34-3.31)	12/199	0.909	3.50 (1.21-9.93)	14/180	0.021	0.90 (0.25-3.20)	14/200	0.86	2.92 (1.51-5.65)	38/382	0.001	0.79 (0.30-2.05)	20/399	0.626
**Skeletal and other**	**GIPC1 Score** high vs. not	1.56 (0.76-3.22)	31/202	0.228	1.07 (0.34-3.31)	12/199	0.909	3.92 (1.58-9.68)	19/180	0.003	0.59 (0.17-2.01)	20/200	0.398	2.37 (1.35-4.16)	50/382	0.003	0.73 (0.34-1.57)	32/399	0.417
**Non-skeletal**	**GIPC1 Score** high vs. not	0.29 (0.10-0.89)	18/202	0.03	0.94 (0.45-1.97)	28/199	0.862	2.20 (0.78-6.18)	15/180	0.135	1.51 (0.62-3.70)	22/200	0.37	0.75 (0.36-1.57)	33/382	0.445	1.20 (0.68-2.12)	50/399	0.526

Supplementary Table 6: Unadjusted and adjusted Cox proportional hazard regressions relating biomarkers and biomarker score and first distant recurrence which has a skeletal component in patients who are pre-menopausal and peri-menopausal. The number of events (n) and total number of patients (N) are presented for each analysis along with the hazard ratio (HR), 95% confidence interval (CI) and p-value (P). Adjusted refers to models which adjust for systemic therapy plan, ER status and lymph node involvement.

			Control					Zoledronic Acid				
			n	N	HR	95% CI	P	n	N	HR	95% CI	P
CAPG	Unadjusted	high vs not	33	248	1.72	(0.85, 3.45)	0.134	15	244	0.76	(0.22, 2.69)	0.670
	Adjusted	high vs not	33	248	1.60	(0.78, 3.26)	0.198	15	244	0.80	(0.22, 2.86)	0.733
GIPC1	Unadjusted	high vs not	39	265	2.52	(1.32, 4.81)	0.005	21	274	0.74	(0.29, 1.91)	0.532
	Adjusted	high vs not	39	265	2.85	(1.47, 5.53)	0.002	21	274	0.68	(0.26, 1.77)	0.430
Biscore	Unadjusted	both high vs not	28	201	3.00	(1.36, 6.63)	0.007	12	194	*		
	Adjusted	both high vs not	28	201	3.47	(1.57, 7.76)	0.002	12	194	*		

*No model estimated due to no events in CAPG and GIPC1 high group (see Supplementary Figure1).

Supplementary Table 7: Unadjusted and adjusted Cox proportional hazard regressions relating biomarkers and biomarker score and first distant recurrence which has a skeletal component in patients who are post-menopausal. The number of events (n) and total number of patients (N) are presented for each analysis along with the hazard ratio (HR), 95% confidence interval (CI) and p-value (P). Adjusted refers to models which adjust for systemic therapy plan, ER status and lymph node involvement.

			Control					Zoledronic Acid				
			n	N	HR	95% CI	P	n	N	HR	95% CI	P
**CAPG**	**Unadjusted**	**high vs not**	11	113	1.47	(0.43, 5.03)	0.538	11	119	2.22	(0.68, 7.27)	0.189
	**Adjusted**	**high vs not**	11	113	1.45	(0.40, 5.28)	0.576	11	119	3.23	(0.92, 11.4)	0.068
**GIPC1**	**Unadjusted**	**high vs not**	11	117	1.62	(0.47, 5.39)	0.433	11	125	0.71	(0.19, 2.68)	0.615
	**Adjusted**	**high vs not**	11	117	1.80	(0.50, 6.57)	0.372	11	125	0.45	(0.11, 1.87)	0.268
**Biscore**	**Unadjusted**	**both high vs not**	8	86	5.47	(1.22, 24.5)	0.026	7	90	0.82	(0.10, 6.87)	0.86
	**Adjusted**	**both high vs not**	8	86	5.92	(1.28, 27.3)	0.023	7	90	0.92	(0.10, 8.38)	0.94

Supplementary Table 8: Unadjusted and adjusted Cox proportional hazard regressions relating biomarkers and biomarker score and overall survival. The number of events (n) and total number of patients (N) are presented for each analysis along with the hazard ratio (HR), 95% confidence interval (CI) and p-value (P). Adjusted refers to models which adjust for systemic therapy plan, ER status and lymph node involvement.

			Control					Zoledronic Acid				
			n	N	HR	95% CI	P	n	N	HR	95% CI	P
CAPG	Unadjusted	high vs not	80	361	1.23	(0.77, 1.97)	0.396	81	363	1.01	(0.62, 1.67)	0.958
	Adjusted	high vs not	80	361	1.38	(0.85, 2.23)	0.192	81	363	1.13	(0.68, 1.87)	0.638
GIPC1	Unadjusted	high vs not	83	382	1.53	(1.00, 2.36)	0.052	98	399	1.28	(0.86, 1.91)	0.228
	Adjusted	high vs not	83	382	1.53	(0.99, 2.36)]	0.056	98	399	1.19	(0.79, 1.79)	0.408
Biscore	Unadjusted	both high vs not	64	287	1.81	(1.01, 3.24)	0.045	68	284	0.71	(0.32, 1.55)	0.385
	Adjusted	both high vs not	64	287	1.74	(0.97, 3.14)	0.064	68	284	0.92	(0.42, 2.04)	0.115

SUPPLEMENTARY FIGURES AND TABLES: LABORATORY DATA

Supplementary Figure 5: Principal component analysis and dendrogram. PC1-v-PC2 clearly shows clustering of the replicates of PCC with GPCC, and also BM1 with BM2, while the LM replicates have clustered separately. The PCA show evidence of the effects of differential normalized volumes relating to, and driving, the separation of the cell types – indicative of underlying cell type-specific differential protein expression. The dendrogram supports and confirms the evidence from the PCA and indicates that the control cells (PCC and GPCC) are closely related while the metastatic cell types (BM1, BM2, LM) are related yet LM separates from BM1 and BM2 (the latter two being very closely related). Colour codes: Black = PCC, Red = GPCC, Green = LM, Dark blue = BM1, Light blue = BM2.

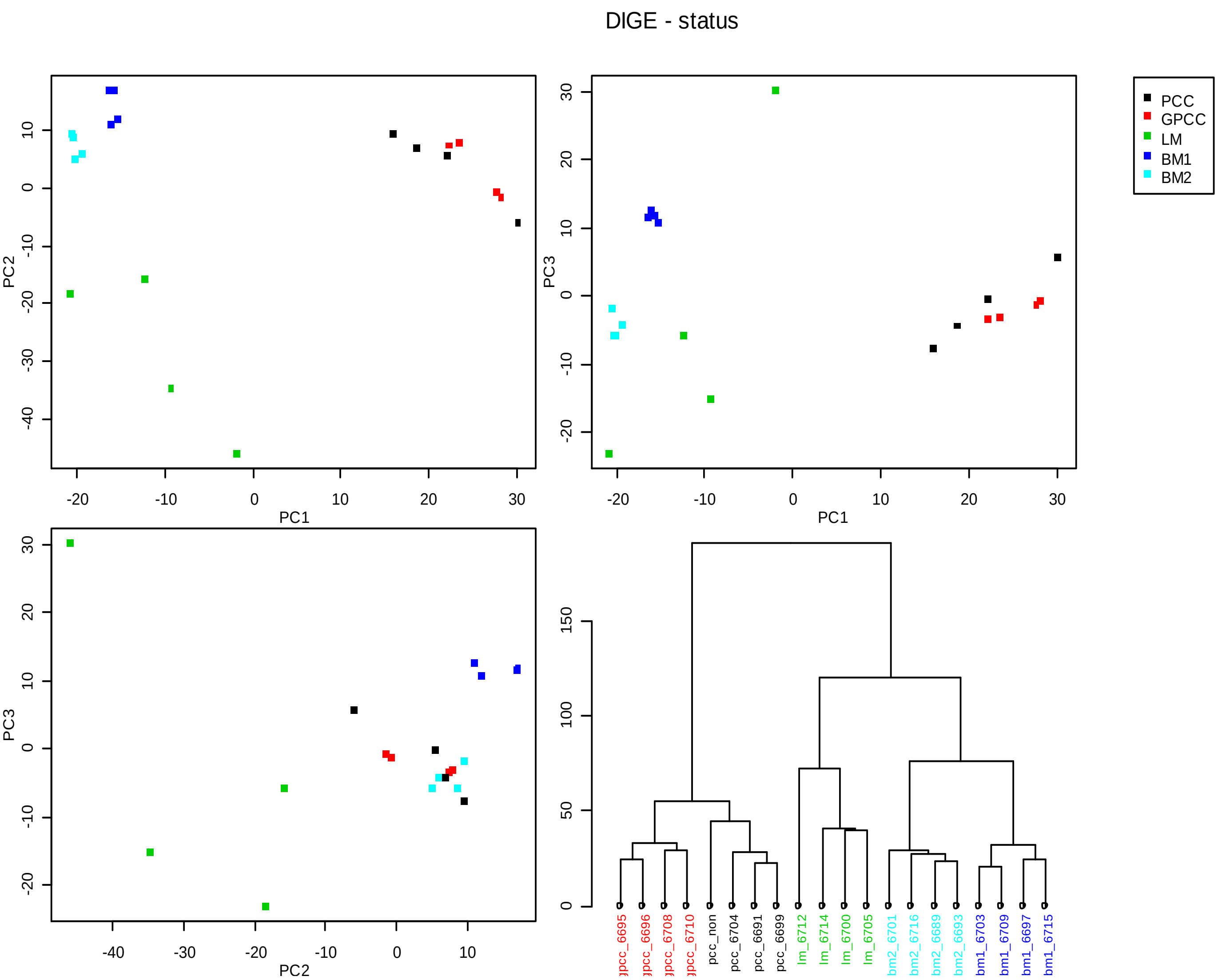

Supplementary Figure 6: Heatmap of 75 unique proteins identified. The normalised volumetric data for the 75 key proteins of interest arising from the 2D-DIGE analysis was used to construct a heatmap indicating the correlations of protein expression, protein identity, and cell type. pcc = MDA-MB-231, gpcc = GFP-tagged MDA-MB-231, lm = lung homing, bm1/bm2 = bone homing (lm, bm1, bm2 = variants of MDA-MB-231).

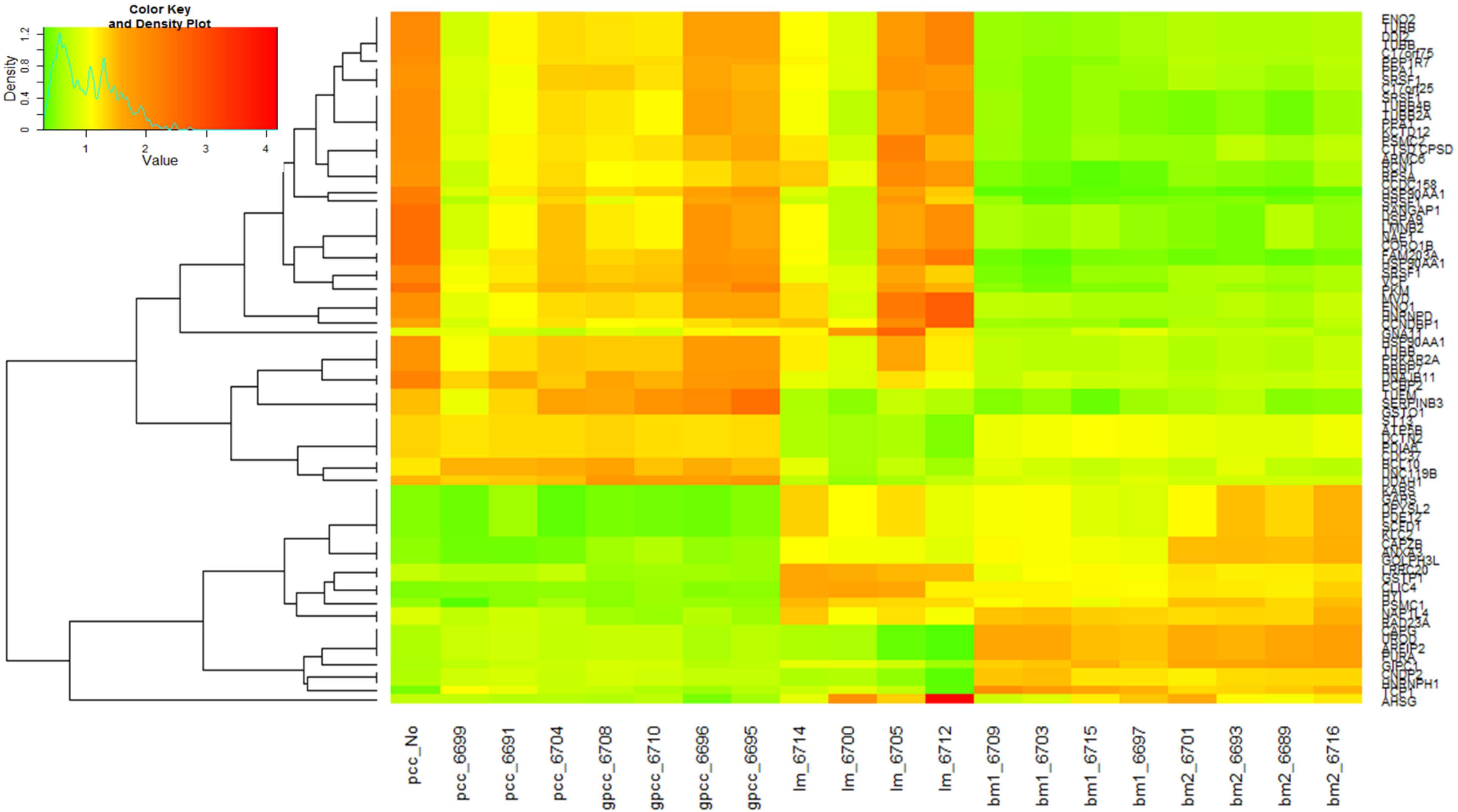

Supplementary Table 9: Brief details of key materials and antibodies used for Western blotting and immunohistochemistry.

Item	Manufacturer/Supplier	Item/Product code
**Western blotting**		
BioRad mini gels (Mini-PROTEAN TGX)	Bio-Rad Laboratories Ltd., Hemel Hempstead, UK	456-1034
1-DE buffer	Sigma-Aldrich, Dorset, UK	S3401
WB buffer	Sigma-Aldrich, Dorset, UK	P2194
Odyssey IR system 2° antibodies	LiCor, Cambridge, UK.	926-68078.
CAPG antibody	Abcam, Cambridge, UK	ab 89511
GIPC1 antibody	Abcam, Cambridge, UK	ab 89684
Colloidal Brilliant Blue	Sigma-Aldrich, Dorset, UK.	B6522
Ponceau red	Sigma-Aldrich, Dorset, UK.	09276-Fluka
**IHC**		
Superfrost Plus slides	BDH, Poole, Dorset	MSS51012WH
Goat serum	Dako UK Ltd, Ely, Cambridgeshire, UK	X0907
HRP-conjugated secondary antibodies	Dako UK Ltd, Ely, Cambridgeshire, UK	P0448
CAPG antibody	Sigma-Aldrich, Dorset, UK	HPA019080
GIPC1 antibody	Abcam, Cambridge, UK	ab89684

Supplementary Table 10: Proteins identified from significantly differentially regulated DIGE gel spots. Qnt. Status indicates the direction of the differential expression and in which cell types: BMs = bone homing cells BM1 +BM2; LM = Lung metastatic cells; METS = BM1+BM2+LM, i.e. all metastatic variants of MDA-MB-231 cell types in the study. KW = p-value derived from Kruskal-Wallis test; FDR = False Discovery Rate; Entry refers to UniProt entry http://www.uniprot.org/.

DIGE Spot#	Qnt. Status	Fold change	KW p-value	FDR q-value	Entry	Protein names	Gene names
751	UP in BMs	2.2	0.004	0.0007	P17987	T-complex protein 1 subunit alpha (TCP-1-alpha) (CCT-alpha)	TCP1 CCT1 CCTA
904	UP in BMs	2.0	0.003	0.0007	P31943	Heterogeneous nuclear ribonucleoprotein H (hnRNP H) [Cleaved into: Heterogeneous nuclear ribonucleoprotein H, N-terminally processed]	HNRNPH1 HNRPH HNRPH1
904	UP in BMs	2.0	0.003	0.0007	Q96KP4	Cytosolic non-specific dipeptidase (EC 3.4.13.18) (CNDP dipeptidase 2) (Glutamate carboxypeptidase-like protein 1) (Peptidase A)	CNDP2 CN2 CPGL PEPA
1106	UP in BMs	2.5	0.003	0.0007	P06132	Uroporphyrinogen decarboxylase (UPD) (URO-D) (EC 4.1.1.37)	UROD
1106	UP in BMs	2.5	0.003	0.0007	P40121	Macrophage-capping protein (Actin regulatory protein CAP-G)	CAPG AFCP MCP
1106	UP in BMs	2.5	0.003	0.0007	P53365	Arfaptin-2 (ADP-ribosylation factor-interacting protein 2) (Partner of RAC1) (Protein POR1)	ARFIP2 POR1
1106	UP in BMs	2.5	0.003	0.0007	Q00577	Transcriptional activator protein Pur-alpha (Purine-rich single-stranded DNA-binding protein alpha)	PURA PUR1
1158	UP in BMs	2.0	0.004	0.0007	O14908	PDZ domain-containing protein GIPC1 (GAIP C-terminus-interacting protein) (RGS-GAIP-interacting protein) (RGS19-interacting protein 1) (Synectin) (Tax interaction protein 2) (TIP-2)	GIPC1 C19orf3 GIPC RGS19IP1
1151	UP in LM	2.1	0.01	0.0008	P29992	Guanine nucleotide-binding protein subunit alpha-11 (G alpha-11) (G-protein subunit alpha-11) (Guanine nucleotide-binding protein G(y) subunit alpha)	GNA11 GA11
692	UP in METS	2.3	0.004	0.0007	P02765	Alpha-2-HS-glycoprotein (Alpha-2-Z-globulin) (Ba-alpha-2-glycoprotein) (Fetuin-A) [Cleaved into: Alpha-2-HS-glycoprotein chain A; Alpha-2-HS-glycoprotein chain B]	AHSG FETUA PRO2743
873	UP in METS	2.0	0.003	0.0007	P54725	UV excision repair protein RAD23 homolog A (HR23A) (hHR23A)	RAD23A
873	UP in METS	2.0	0.003	0.0007	Q99733	Nucleosome assembly protein 1-like 4 (Nucleosome assembly protein 2) (NAP-2)	NAP1L4 NAP2
1015	UP in METS	2.6	0.002	0.0007	P62191	26S protease regulatory subunit 4 (P26s4) (26S proteasome AAA-ATPase subunit RPT2) (Proteasome 26S subunit ATPase 1)	PSMC1
1332	UP in METS	2.4	0.002	0.0007	P12429	Annexin A3 (35-alpha calcimedin) (Annexin III) (Annexin-3) (Inositol 1,2-cyclic phosphate 2-phosphohydrolase) (Lipocortin III) (Placental anticoagulant protein III) (PAP-III)	ANXA3 ANX3
1332	UP in METS	2.4	0.002	0.0007	P47756	F-actin-capping protein subunit beta (CapZ beta)	CAPZB
1332	UP in METS	2.4	0.002	0.0007	Q9H4A5	Golgi phosphoprotein 3-like (GPP34-related protein)	GOLPH3L GPP34R
1432	UP in METS	2.6	0.003	0.0007	Q5T013	Putative hydroxypyruvate isomerase (EC 5.3.1.22) (Endothelial cell apoptosis protein E-CE1)	HYI HT036 SB156
1432	UP in METS	2.6	0.003	0.0007	Q9Y696	Chloride intracellular channel protein 4 (Intracellular chloride ion channel protein p64H1)	CLIC4
1537	UP in METS	2.1	0.001	0.0007	P09211	Glutathione S-transferase P (EC 2.5.1.18) (GST class-pi) (GSTP1-1)	GSTP1 FAEES3 GST3

DIGE Spot#	Qnt. Status	Fold change	KW p-value	FDR q-value	Entry	Protein names	Gene names
1537	UP in METS	2.1	0.001	0.0007	Q8TCA0	Leucine-rich repeat-containing protein 20	LRRC20 UNQ2429/PRO4989
1872	UP in METS	2.7	0.003	0.0007	P41250	Glycine--tRNA ligase (EC 6.1.1.14) (Diadenosine tetraphosphate synthetase) (AP-4-A synthetase) (Glycyl-tRNA synthetase) (GlyRS)	GARS
1872	UP in METS	2.7	0.003	0.0007	Q15046	Lysine--tRNA ligase (EC 6.1.1.6) (Lysyl-tRNA synthetase) (LysRS)	KARS KIAA0070
1872	UP in METS	2.7	0.003	0.0007	Q59GB4	Dihydropyrimidinase-like 2 variant (Fragment)	DPYSL2
1872	UP in METS	2.7	0.003	0.0007	Q6L8Q7	2',5'-phosphodiesterase 12 (2'-PDE) (2-PDE) (EC 3.1.4.-) (Mitochondrial deadenylase) (EC 3.1.13.4)	PDE12
1872	UP in METS	2.7	0.003	0.0007	Q8WVM8	Sec1 family domain-containing protein 1 (SLY1 homolog) (Sly1p) (Syntaxin-binding protein 1-like 2)	SCFD1 C14orf163 KIAA0917 STXBP1L2 FKSG23
1872	UP in METS	2.7	0.003	0.0007	Q9H0B6	Kinesin light chain 2 (KLC 2)	KLC2
716	DOWN in BMs	2.6	0.007	0.0008	P38646	Stress-70 protein, mitochondrial (75 kDa glucose-regulated protein) (GRP-75) (Heat shock 70 kDa protein 9) (Mortalin) (MOT) (Peptide-binding protein 74) (PBP74)	HSPA9 GRP75 HSPA9B mt-HSP70
716	DOWN in BMs	2.6	0.007	0.0008	P46060	Ran GTPase-activating protein 1 (RanGAP1)	RANGAP1 KIAA1835 SD
716	DOWN in BMs	2.6	0.007	0.0008	Q03252	Lamin-B2	LMNB2 LMN2
716	DOWN in BMs	2.6	0.007	0.0008	Q13564	NEDD8-activating enzyme E1 regulatory subunit (Amyloid beta precursor protein-binding protein 1, 59 kDa) (APP-BP1) (Amyloid protein-binding protein 1) (Proto-oncogene protein 1)	NAE1 APPBP1 HPP1
716	DOWN in BMs	2.6	0.007	0.0008	Q9BR76	Coronin-1B (Coronin-2)	CORO1B
939	DOWN in BMs	2.0	0.005	0.0007	P07437	Tubulin beta chain (Tubulin beta-5 chain)	TUBB TUBB5 OK/SW-cl.56
939	DOWN in BMs	2.0	0.005	0.0007	P07900	Heat shock protein HSP 90-alpha (Heat shock 86 kDa) (HSP 86) (HSP86) (Lipopolysaccharide-associated protein 2) (LAP-2) (LPS-associated protein 2) (Renal carcinoma antigen NY-REN-38)	HSP90AA1 HSP90A HSPC1 HSPCA
939	DOWN in BMs	2.0	0.005	0.0007	P13861	cAMP-dependent protein kinase type II-alpha regulatory subunit	PRKAR2A PKR2 PRKAR2
939	DOWN in BMs	2.0	0.005	0.0007	Q16576	Histone-binding protein RBBP7 (Histone acetyltransferase type B subunit 2) (Nucleosome-remodeling factor subunit RBAP46) (Retinoblastoma-binding protein 7) (RBBP-7) (Retinoblastoma-binding protein p46)	RBBP7 RBAP46
952	DOWN in BMs	3.8	0.008	0.0008	P07900	Heat shock protein HSP 90-alpha (Heat shock 86 kDa) (HSP 86) (HSP86)	HSP90AA1

						(Lipopolysaccharide-associated protein 2) (LAP-2) (LPS-associated protein 2) (Renal carcinoma antigen NY-REN-38)	HSP90A HSPC1 HSPCA
**DIGE Spot#**	**Qnt. Status**	**Fold change**	**KW p-value**	**FDR q-value**	**Entry**	**Protein names**	**Gene names**
952	DOWN in BMs	3.8	0.008	0.0008	Q9BTY7	Protein FAM203A (Brain protein 16)	FAM203A BRP16 C8orf30A
978	DOWN in BMs	2.4	0.005	0.0007	P07437	Tubulin beta chain (Tubulin beta-5 chain)	TUBB TUBB5 OK/SW-cl.56
978	DOWN in BMs	2.4	0.005	0.0007	P09104	Gamma-enolase (EC 4.2.1.11) (2-phospho-D-glycerate hydro-lyase) (Enolase 2) (Neural enolase) (Neuron-specific enolase) (NSE)	ENO2
978	DOWN in BMs	2.4	0.005	0.0007	Q5TDH0	Protein DDI1 homolog 2	DDI2
978	DOWN in BMs	2.4	0.005	0.0007	Q6LC01	MRNA encoding beta-tubulin. (from clone D-beta-1) (Fragment)	TUBB
978	DOWN in BMs	2.4	0.005	0.0007	Q9HAS0	Protein Njmu-R1	C17orf75
1005	DOWN in BMs	2.3	0.007	0.0007	P07339	Cathepsin D (EC 3.4.23.5) [Cleaved into: Cathepsin D light chain; Cathepsin D heavy chain]	CTSD CPSD
1005	DOWN in BMs	2.3	0.007	0.0007	P35998	26S protease regulatory subunit 7 (26S proteasome AAA-ATPase subunit RPT1) (Proteasome 26S subunit ATPase 2) (Protein MSS1)	PSMC2 MSS1
1005	DOWN in BMs	2.3	0.007	0.0007	Q6NXE6	Armadillo repeat-containing protein 6	ARMC6
1032	DOWN in BMs	2.4	0.005	0.0007	Q15435	Protein phosphatase 1 regulatory subunit 7 (Protein phosphatase 1 regulatory subunit 22)	PPP1R7 SDS22
1071	DOWN in BMs	2.3	0.007	0.0008	P06733	Alpha-enolase (EC 4.2.1.11) (2-phospho-D-glycerate hydro-lyase) (C-myc promoter-binding protein) (Enolase 1) (MBP-1) (MPB-1) (Non-neural enolase) (NNE) (Phosphopyruvate hydratase) (Plasminogen-binding protein)	ENO1 ENO1L1 MBPB1 MPB1
1071	DOWN in BMs	2.3	0.007	0.0008	P53602	Diphosphomevalonate decarboxylase (EC 4.1.1.33) (Mevalonate (diphospho)decarboxylase) (MDDase) (Mevalonate pyrophosphate decarboxylase)	MVD MPD
1071	DOWN in BMs	2.3	0.007	0.0008	Q14103	Heterogeneous nuclear ribonucleoprotein D0 (hnRNP D0) (AU-rich element RNA-binding protein 1)	HNRNPD AUF1 HNRPD
1180	DOWN in BMs	3.0	0.004	0.0007	P14618	Pyruvate kinase PKM (EC 2.7.1.40) (Cytosolic thyroid hormone-binding protein) (CTHBP) (Opa-interacting protein 3) (OIP-3) (Pyruvate kinase 2/3) (Pyruvate kinase muscle isozyme) (Thyroid hormone-binding protein 1) (THBP1) (Tumor M2-PK) (p58)	PKM OIP3 PK2 PK3 PKM2
1254	DOWN in BMs	2.7	0.008	0.0008	P68371	Tubulin beta-4B chain (Tubulin beta-2 chain) (Tubulin beta-2C chain)	TUBB4B TUBB2C
1254	DOWN in BMs	2.7	0.008	0.0008	Q07955	Serine/arginine-rich splicing factor 1 (Alternative-splicing factor 1) (ASF-1) (Splicing factor, arginine/serine-rich 1) (pre-mRNA-splicing factor SF2, P33 subunit)	SRSF1 ASF SF2 SF2P33 SFRS1 OK/SW-cl.3
1254	DOWN in BMs	2.7	0.008	0.0008	Q13885	Tubulin beta-2A chain (Tubulin beta class IIa)	TUBB2A TUBB2
1254	DOWN in BMs	2.7	0.008	0.0008	Q15181	Inorganic pyrophosphatase (EC 3.6.1.1) (Pyrophosphate phospho-hydrolase) (PPase)	PPA1 IOPPP PP
1254	DOWN in BMs	2.7	0.008	0.0008	Q96CX2	BTB/POZ domain-containing protein KCTD12 (Pfetin) (Predominantly fetal expressed T1 domain)	KCTD12 C13orf2 KIAA1778 PFET1

1267	DOWN in BMs	4.0	0.006	0.0007	P07900	Heat shock protein HSP 90-alpha (Heat shock 86 kDa) (HSP 86) (HSP86) (Lipopolysaccharide-associated protein 2) (LAP-2) (LPS-associated protein 2) (Renal carcinoma antigen NY-REN-38)	HSP90AA1 HSP90A HSPC1 HSPCA
**DIGE Spot#**	**Qnt. Status**	**Fold change**	**KW p-value**	**FDR q-value**	**Entry**	**Protein names**	**Gene names**
1286	DOWN in BMs	2.4	0.005	0.0007	Q07955	Serine/arginine-rich splicing factor 1 (Alternative-splicing factor 1) (ASF-1) (Splicing factor, arginine/serine-rich 1) (pre-mRNA-splicing factor SF2, P33 subunit)	SRSF1 ASF SF2 SF2P33 SFRS1 OK/SW-cl.3
1286	DOWN in BMs	2.4	0.005	0.0007	Q15181	Inorganic pyrophosphatase (EC 3.6.1.1) (Pyrophosphate phospho-hydrolase) (PPase)	PPA1 IOPPP PP
1286	DOWN in BMs	2.4	0.005	0.0007	Q9Y3E8	CGI-150 protein	C17orf25
1288	DOWN in BMs	2.6	0.003	0.0007	P55072	Transitional endoplasmic reticulum ATPase (TER ATPase) (EC 3.6.4.6) (15S Mg(2+)-ATPase p97 subunit) (Valosin-containing protein) (VCP)	VCP
1288	DOWN in BMs	2.6	0.003	0.0007	Q07955	Serine/arginine-rich splicing factor 1 (Alternative-splicing factor 1) (ASF-1) (Splicing factor, arginine/serine-rich 1) (pre-mRNA-splicing factor SF2, P33 subunit)	SRSF1 ASF SF2 SF2P33 SFRS1 OK/SW-cl.3
1294	DOWN in BMs	2.9	0.007	0.0008	Q07955	Serine/arginine-rich splicing factor 1 (Alternative-splicing factor 1) (ASF-1) (Splicing factor, arginine/serine-rich 1) (pre-mRNA-splicing factor SF2, P33 subunit)	SRSF1 ASF SF2 SF2P33 SFRS1 OK/SW-cl.3
1832	DOWN in BMs	2.3	0.005	0.0007	O95273	Cyclin-D1-binding protein 1 (Grap2 and cyclin-D-interacting protein) (Human homolog of Maid)	CCNDBP1 DIP1 GCIP HHM
1833	DOWN in BMs	2.9	0.005	0.0007	P08865	40S ribosomal protein SA (37 kDa laminin receptor precursor) (37LRP) (37/67 kDa laminin receptor) (LRP/LR) (67 kDa laminin receptor) (67LR) (Colon carcinoma laminin-binding protein) (Laminin receptor 1) (LamR) (Laminin-binding protein precursor p40) (LBP/p40) (Multidrug resistance-associated protein MGr1-Ag) (NEM/1CHD4)	RPSA LAMBR LAMR1
1833	DOWN in BMs	2.9	0.005	0.0007	Q15293	Reticulocalbin-1	RCN1 RCN
1833	DOWN in BMs	2.9	0.005	0.0007	Q5M9N0	Coiled-coil domain-containing protein 158	CCDC158
940	DOWN in LM	2.0	0.002	0.0007	P06576	ATP synthase subunit beta, mitochondrial (EC 3.6.3.14)	ATP5B ATPMB ATPSB
940	DOWN in LM	2.0	0.002	0.0007	P50502	Hsc70-interacting protein (Hip) (Aging-associated protein 2) (Progesterone receptor-associated p48 protein) (Protein FAM10A1) (Putative tumor suppressor ST13) (Renal carcinoma antigen NY-REN-33) (Suppression of tumorigenicity 13 protein)	ST13 AAG2 FAM10A1 HIP SNC6
940	DOWN in LM	2.0	0.002	0.0007	Q13561	Dynactin subunit 2 (50 kDa dynein-associated polypeptide) (Dynactin complex 50 kDa subunit) (DCTN-50) (p50 dynamitin)	DCTN2 DCTN50
940	DOWN in LM	2.0	0.002	0.0007	Q15084	Protein disulfide-isomerase A6 (EC 5.3.4.1) (Endoplasmic reticulum protein 5) (ER protein 5) (ERp5) (Protein disulfide isomerase P5) (Thioredoxin domain-containing protein 7)	PDIA6 ERP5 P5 TXNDC7
940	DOWN in LM	2.0	0.002	0.0007	Q16543	Hsp90 co-chaperone Cdc37 (Hsp90 chaperone protein kinase-targeting subunit) (p50Cdc37) [Cleaved into: Hsp90 co-chaperone Cdc37, N-terminally processed]	CDC37 CDC37A

1095	DOWN in METS	2.0	0.002	0.0007	Q15366	Poly(rC)-binding protein 2 (Alpha-CP2) (Heterogeneous nuclear ribonucleoprotein E2) (hnRNP E2)	PCBP2

DIGE Spot#	Qnt. Status	Fold change	KW p-value	FDR q-value	Entry	Protein names	Gene names
1095	DOWN in METS	2.0	0.002	0.0007	Q9UBS4	DnaJ homolog subfamily B member 11 (APOBEC1-binding protein 2) (ABBP-2) (DnaJ protein homolog 9) (ER-associated DNAJ) (ER-associated Hsp40 co-chaperone) (Endoplasmic reticulum DNA J domain-containing protein 3) (ER-resident protein ERdj3) (ERdj3) (ERj3p) (HEDJ) (Human DnaJ protein 9) (hDj-9) (PWP1-interacting protein 4)	DNAJB11 EDJ ERJ3 HDJ9 PSEC0121 UNQ537/PRO1080
1203	DOWN in METS	2.1	0.002	0.0007	O94760	N(G),N(G)-dimethylarginine dimethylaminohydrolase 1 (DDAH-1) (Dimethylarginine dimethylaminohydrolase 1) (EC 3.5.3.18) (DDAHI) (Dimethylargininase-1)	DDAH1 DDAH
1336	DOWN in METS	2.9	0.003	0.0007	P29508	Serpin B3 (Protein T4-A) (Squamous cell carcinoma antigen 1) (SCCA-1)	SERPINB3 SCCA SCCA1
1336	DOWN in METS	2.9	0.003	0.0007	P49411	Elongation factor Tu, mitochondrial (EF-Tu) (P43)	TUFM
1336	DOWN in METS	2.9	0.003	0.0007	P78417	Glutathione S-transferase omega-1 (GSTO-1) (EC 2.5.1.18) (Glutathione S-transferase omega 1-1) (GSTO 1-1) (Glutathione-dependent dehydroascorbate reductase) (EC 1.8.5.1) (Monomethylarsonic acid reductase) (MMA(V) reductase) (EC 1.20.4.2) (S-(Phenacyl)glutathione reductase) (SPG-R)	GSTO1 GSTTLP28
1383	DOWN in METS	2.0	0.007	0.0007	A6NIH7	Protein unc-119 homolog B	UNC119B
1383	DOWN in METS	2.0	0.007	0.0007	O95999	B-cell lymphoma/leukemia 10 (B-cell CLL/lymphoma 10) (Bcl-10) (CARD-containing molecule enhancing NF-kappa-B) (CARD-like apoptotic protein) (hCLAP) (CED-3/ICH-1 prodomain homologous E10-like regulator) (CIPER) (Cellular homolog of vCARMEN) (cCARMEN) (Cellular-E10) (c-E10) (Mammalian CARD-containing adapter molecule E10) (mE10)	BCL10 CIPER CLAP

*Proteins identified from multiple DIGE gel spots

Protein identifier	DIGE spot#	Gel-resolved MW (kDa)	Gel-resolved pI
P07900	939	43.7	4.9
	952	43.5	4.8
	1267	35.7	5.2
Q15181	1254	35.0	5.4
	1286	36.4	5.4
Q07955	1254	35.0	5.5
	1286	34.6	5.1
	1288	35.0	5.4
	1294	36.4	5.4
P07437	939	43.0	5.0
	978	36.4	5.4

References:

1. Hornigold N, Craven RA, Keen NJ et al. Upregulation of Hic-5 in glomerulosclerosis and its regulation of mesangial cell apoptosis. Kidney Int 2010;77:329-38.
2. Simpkins SA, Hanby AM, Holliday DL et al. Clinical and functional signifciance of loss of vaeolin-1 expression in breast cancer-associated fibroblasts. J Pathol 2012;227:490-8.
3. Kang Y. Siegel PM. Shu W et al. A multigenic programme mediating breast cancer metastasis to bone. Cancer Cell 2003;3:537-549.
4. Minn AJ, Gupta GP, Siegel PM, et al. Genes that mediate breast cancer metastasis to lung. Nature 2005;436:518-24.
5. Ramagli, LS. Quantifying protein in 2-D PAGE solubilization buffers. Methods in Molecular Biology, 1999;112: 99-103.
6. Bradford, MM. A rapid and sensitive method for the quantitation of microgram quantities of protein utilizing the principle of protein-dye binding. Anal Biochem 1976;72:248-54.
7. R Development Core Team. R: A language and environment for statistical computing. R Foundation for Statistical Computing, Vienna, Austria. ISBN 3-900051-07-0, 2008; URL http://www.R-project.org
8. http://www.rstudio.com/
9. Siegel, SC. Nonparametric statistics for the behavioral sciences 1998; McGraw-Hill, New York, London.
10. Storey J. A direct approach to false discovery rates. J Royal Statistical Soc Series B-Statistical Methodology 2002;64:479-498.
11. Benjamini Y, Drai D, Elmerr G et al. Controlling the false discovery rate in behavior genetics research. Behav Brain Res 2001;125:279-84.
12. Laemmli UK. Cleavage of structural proteins during the assembly of the head of bacteriophage T4. Nature 1970;227:680-5.
13. Ferguson RE, Carroll HP, Harris A et al. Housekeeping proteins: a preliminary study illustrating some limitations as useful references in protein expression studies. Proteomics 2005;5:566-71.
14. Nomura H, Uzawak K, Ishigami T et al. Clinical significance of gelsolin-like actin-capping protein expression in oral carcinogenesis: an immunohistochemical study of premalignant and malignant lesions of the oral cavity. BMC Cancer 2008;8:39.
15. Van Ginkel PR, Gee RL, Walker TM et al. The identification and differential expression of calcium-binding proteins associated with ocular melanoma. Biochim Biophys Acta 1998;1448:290-7.
16. Glaser J, Neumann MH, Mei Q et al. Macrophage Capping Protein CapG is a putative oncogene involved in migration and invasiveness in ovarian carcinoma. Biomed Res Int 2014;epub Apr 2.
17. Thompson CC, Ashcroft fj, Patel S et al. Pancreatic cancer cells overexpress gelsolin family-capping proteins, which contribute to their cell motility. Gut 2007; 56:95-106.
18. Renz M, Betz B, Viedersacher D et al. Invasive breast cancer cells exhibit increased mobility of the actin-binding protein CapG. Int J Cancer 2008; 122:1476-82.
19. Renz M, Langowski J. Dynamics of the CapG actin-binding protein in the cell nucleus studied by FRAP and FCS. Chromosome Res 2008;16:427-37.

20. Kang S, Kim MJ, An H et al. Proteomic molecular portrait of interface zone in breast cancer. J Proteome Res 2010;9:5638-45.
21. Van Impe K, Bethuyne J, Cool S et al. A nanobody targeting the F-actin capping protein CapG restrains breast cancer metastasis. Breast Cancer Res, 2013;15:R116.
22. Varsano T, Taupin V, Guo L et al. The PDZ protein GIPC regulates trafficking of the LPA1 receptor from APPL signaling endosomes and attenuates the cell's response to LPA. PLoS One 2012;**7**:49227.
23. Lou X, Yano H, Lee F et al. GIPC and GAIP form a complex with TrkA: a putative link between G protein and receptor tyrosine kinase pathways. Mol Biol Cell 2001;12:615-27.
24. Varsano T, Dong MQ, Niesman I et al. GIPC is recruited by APPL to peripheral TrkA endosomes and regulates TrkA trafficking and signaling. Mol Cell Biol 2006;26:8942-52.
25. Lanahan AA, Hermans K, Claes F et al. VEGF receptor 2 endocytic trafficking regulates arterial morphogenesis. Dev Cell 2010;18:713-24.
26. Townsend TA, Robinson JY, How T et al. Endocardial cell epithelial-mesenchymal transformation requires Type III TGFbeta receptor interaction with GIPC. Cell Signal 2012;24:247-56.
27. Jeanneteau F, Diaz J, Sokoloff P et al. Interactions of GIPC with dopamine D2, D3 but not D4 receptors define a novel mode of regulation of G protein-coupled receptors. Mol Biol Cell 2004;15:696-705.
28. Hirakawa T, Galet C, Kishi M et al. GIPC binds to the human lutropin receptor (hLHR) through an unusual PDZ domain binding motif, and it regulates the sorting of the internalized human choriogonadotropin and the density of cell surface hLHR. J Biol Chem 2003;278:49348-57.
29. Lee NY, Ray B, How T et al. Endoglin promotes transforming growth factor beta-mediated Smad 1/5/8 signaling and inhibits endothelial cell migration through its association with GIPC. J Biol Chem 2008;283:32527-33.
30. Lee JD, Hempel N, Lee NY et al. The type III TGF-beta receptor suppresses breast cancer progression through GIPC-mediated inhibition of TGF-beta signaling. Carcinogenesis 2010;31:175-83.
31. Wang L, Lau JS, Patra CR et al. RGS-GAIP-interacting protein controls breast cancer progression. Mol Cancer Res 2010;8:1591-600.
32. Wu D, Haruta A, Wei Q. GIPC1 interacts with MyoGEF and promotes MDA-MB-231 breast cancer cell invasion. J Biol Chem 2010;285:28643-50.
